# Competition and predation as possible causes of bacterial rarity

**DOI:** 10.1111/1462-2920.14569

**Published:** 2019-03-18

**Authors:** Viola Kurm, Wim H. van der Putten, Simone Weidner, Stefan Geisen, Basten L. Snoek, Tanja Bakx, Wilhelmina H. Gera Hol

**Affiliations:** ^1^ Department of Terrestrial Ecology Netherlands Institute of Ecology (NIOO‐KNAW) P.O. Box 50, 6700 AB, Wageningen The Netherlands; ^2^ Laboratory of Nematology Wageningen University P.O. Box 8123, 6700 ES, Wageningen The Netherlands; ^3^ Institute of Environmental Biology, Ecology and Biodiversity Utrecht University Padualaan 8, 3584 CH, Utrecht The Netherlands; ^4^ Theoretical Biology and Bioinformatics Utrecht University Padualaan 8, 3584 CH, Utrecht The Netherlands

## Abstract

We assembled communities of bacteria and exposed them to different nutrient concentrations with or without predation by protists. Taxa that were rare in the field were less abundant at low nutrient concentrations than common taxa, independent of predation. However, some taxa that were rare in the field became highly abundant in the assembled communities, especially under ample nutrient availability. This high abundance points at a possible competitive advantage of some rare bacterial taxa under nutrient‐rich conditions. In contrast, the abundance of most rare bacterial taxa decreased at low resource availability. Since low resource availability will be the prevailing situation in most soils, our data suggests that under those conditions poor competitiveness for limiting resources may contribute to bacterial rarity. Interestingly, taxa that were rare in the field and most successful under predator‐free conditions in the lab also tended to be more reduced by predation than common taxa. This suggests that predation contributes to rarity of bacterial taxa in the field. We further discuss whether there may be a trade‐off between competitiveness and predation resistance. The substantial variability among taxa in their responses to competition and predation suggests that other factors, for example abiotic conditions and dispersal ability, also influence the local abundance of soil bacteria.

## Introduction

Most communities consist of only a few abundant species, whereas the majority of species are low in abundance, or rare (Magurran and Henderson, 2003). This pattern, which applies to organisms of any size, from macroorganisms to microorganisms, has stimulated studies addressing the question why so many species are rare (see e.g. Torsvik *et al*., 1996; Buckling *et al*., 2000). However, few empirical studies elucidate possible causes of microbial rarity. Although, several studies have been carried out to examine how single factors affect the abundance of microbes (Velicer and Lenski, 1999; Bouvier and Del Giorgio, 2007), little is known about combined effects of such factors. For example, effects of resource availability on species abundance will depend on their physiological, morphological or behavioural characteristics or traits (Martiny *et al*., 2015), such as growth rate, the use of specific resources and reproductive capacity (Flather and Sieg, 2007). Slow‐growing species (Murray *et al*., 2002) and species with a highly specialized niche (Wamelink *et al*., 2014) are supposed to be less abundant than fast‐growing species. The consideration of single traits may not be sufficient to explain species abundance in the field. Further, it is not well understood how species with different trait combinations may perform under competition with or without predators, when varying resource availabilities.

Competition for nutrients can substantially influence species abundance (Tilman *et al*., 1982). The competitive success of a species can be partially predicted by certain species traits, such as maximal growth rate. For example, fast‐growing species may be good competitors and become abundant by quickly exploiting resources (Grime, 1977; Amarasekare, 2003). However, a fast maximal growth rate does not always lead to high abundance in more complex communities, especially under low resource availability (Grover, 1991; Aerts, 1999). Therefore, nutrient availability, in addition to growth rate, may interact to determine competitive success and, consequently, species abundances. Slow‐growing species often effectively use low concentrations of nutrients (Harpole and Tilman, 2006). In addition, the R* theory (Tilman, 1982) states that a species with the ability to reduce a limiting resource to the lowest level will be able to outcompete all other species. According to this theory, slow‐growing species that can continue taking up nutrients under low resource availability should be able to outcompete fast‐growing species under low resource availability, while species with fast maximal growth rates should have an advantage when resource availability is high. Thus, considering the growth rate of contrasting species in concert with nutrient availability might enhance predictability of abundance in the community.

Many ecological concepts that have been developed for macroorganisms can be applied to the microbial world as well (Barberán *et al*., 2014), meaning that the same traits or trait combinations might predict the competitive success of microorganisms. For example, bacterial taxa [this term is preferred over ‘species’ due to incongruence in the bacterial species concept (Rosselló‐Mora and Amann, 2001; Rosselló‐Móra and Amann, 2015)] may also be differentiated into fast‐ (i.e. copiotrophs) and slow‐growing (i.e. oligotrophs) respectively (Fierer *et al*., 2007). Results from *in vitro* as well as *in situ* studies suggest that slow‐growing taxa may have an advantage under low nutrient concentrations due to their higher substrate affinities (Suwa and Hattori, 1984; Vergin *et al*., 2013). Therefore, nutrient availability might influence the outcome of competition for nutrients between copiotrophic and oligotrophic bacteria. However, performance in the presence of competitors and nutrient status alone might not sufficiently explain field abundance, as many rare bacterial taxa are able to grow rapidly and outcompete taxa that are more abundant in the field (Campbell *et al*., 2011; Baldrian *et al*., 2012).

Other key factors that may influence taxon abundances are predation and viral lysis, which both can alter the outcome of competitive interactions. The kill‐the‐winner hypothesis, coined by Thingstad and Lignell (1997), proposes that the most competitive taxon will also be suppressed most by predation. Disproportional predation on the most competitive taxon might be the result of a chance encounter (Bouvier and Del Giorgio, 2007), but it may as well be that certain taxa have become specialized by investing more energy in traits that increase their competitiveness, rather than their resistance or tolerance to predators (Herms and Mattson, 1992; Strauss and Agrawal, 1999; Winter *et al*., 2010). Consequently, this differential allocation of limiting resources may result in contrasting performances of a taxon in communities with versus without predators. Such a trade‐off between competitiveness and defence may lead to resistant taxa outperforming nonresistant taxa in the presence of predators, while in the absence of predators, the nonresistant taxa can become dominant.

Protists are among the key predators of bacteria (Geisen *et al*., 2018). Bacterial resistance mechanisms against predation by protists include morphological defences, such as the formation of filaments or cell aggregates, and chemical defences, such as toxin production (Jürgens and Matz, 2002). The cost of such anti‐protist defences, and hence, the magnitude of a trade‐off with bacterial competitiveness, is poorly understood. Different types of defences might incur different fitness costs and, therefore, variations in strength of such a trade‐off might arise (Bohannan and Lenski, 2000a). It has been shown that taxa with rapid growth rates and high competitive ability can be reduced to low abundances under predation by protists, suggesting that predation may contribute to bacterial rarity (Neuenschwander *et al*., 2015; Batani *et al*., 2016). However, it is still unclear if this phenomenon of increased predation on fast‐growing taxa is due to a trade‐off between fast growth and predation resistance. In addition, it is not clear if differences in this trade‐off might be related to taxon abundance. Taxa that experience fitness costs due to anti‐predator defences can be expected to be less common than taxa with lower defence costs. Alternatively, chance of encounter with a predator could determine intensity of predation. It has been suggested that low abundance protects bacteria against predation and viral lysis (Galand *et al*., 2009). Hence, the role of protist predation as a factor determining bacterial abundances is still poorly understood.

Several studies have shown that nutrient availability can alter the relative importance of competition for nutrients and predation in determining bacterial population dynamics. In nutrient‐poor environments, competition for nutrients was the most important factor shaping species' abundances in plant and rocky shoreline communities (Proulx and Mazumder, 1998; Worm *et al*., 2002). However, in both cases at high nutrient levels consumption by herbivores or predators ultimately was the factor that shaped community composition. The resource availability hypothesis (Endara and Coley, 2011) predicts that slow‐growing plant species are not only more competitive under low nutrient concentrations, but also better defended against herbivores. Protection of tissues of slow‐growing plant species is essential as the cost of their tissue loss is relatively high (Herms and Mattson 1992). In the bacterial world, competition and predation can also be modified by nutrient availability (Bohannan and Lenski, 2000b; Hahn and Höfle, 2001; Geisen *et al*., 2018). Grazing‐resistant forms of bacteria have been found to occur mostly under nutrient‐rich conditions, indicating a high cost of predation resistance (Jürgens and Matz, 2002; Corno and Jürgens, 2008).

The aim of the present study was to investigate how nutrient availability can influence competition and predation of bacterial taxa with low and high potential growth rates. In addition, these capacities will be linked to their abundance in the field. Based on the oligotrophic–copiotrophic concept, we expected fast‐growing taxa to have competitive advantage under high, but not under low nutrient availability. Thus, we tested the hypothesis that in the absence of predators, taxa with high potential growth rates will become relatively abundant under high nutrient concentrations, whereas taxa with low maximal growth rates will become relatively abundant under low nutrient concentrations (1). In order to explain why taxa of equal activity and growth rate may be either rare or common in the field, we tested the hypothesis that predation affects rare taxa more negatively than common ones (2) and that taxon abundance depends on an interaction between predation and nutrient availability (3). To test these hypotheses we used bacterial isolates, which we grouped into slow‐ and fast‐growing taxa based on previous assessments of their abundance in the field and potential growth rate *in vitro* (Kurm *et al*., 2017) (Table 1). We defined four taxon categories: rare in the field and slow‐growing (*Rare/Slow*), common in the field and slow‐growing (*Common/Slow*), rare in the field and fast‐growing (*Rare/Fast*) and common in the field and fast‐growing (*Common/Fast*) taxa, which is similar to the categorization made by Newton and Shade (2016). We studied their abundances in constructed communities at four different nutrient levels with and without predation by protists.

**Table 1:** Description of the bacterial isolates used in this study; relative abundance the field and maximum growth rate as determined in Kurm and colleagues (2017).

Taxon	Family	Genus	Relative abundance in the field%	Maximum growth rate TSB (h^−1^)	Abundance group	Growth group
*S1*	Methylobacteriaceae	*Methylobacterium*	0.000272	0.0069	rare	slow
*S2*	Carnobacteriaceae	*Carnobacterium*	0.000470	0.0344	rare	slow
*S3*	Sphingomonadaceae	*Sphingomonas*	0.000000	0.0371	rare	slow
*S4*	Comamonadaceae		0.000000	0.0383	rare	slow
*S5*	Phyllobacteriaceae	*Mesorhizobium*	0.000000	0.0306	rare	slow
*S6*	Microbacteriaceae	*Curtobacterium*	0.000000	0.0834	rare	slow
*S7*	Caulobacteraceae	*Phenylobacterium*	0.089863	0.0102	common	slow
*S8*	Kineosporiaceae	*Quadrisphaera*	0.109113	0.0856	common	slow
*S9*	Nocardioidaceae		0.001160	0.0592	common	slow
*S10*	Xanthobacteraceae		0.001970	0.0106	common	slow
*S11*	Bradyrhizobiaceae	*Bosea*	0.019739	0.0273	common	slow
*S12*	Phyllobacteriaceae	*Mesorhizobium*	0.012511	0.0628	common	slow
*S13*	Staphylococcaceae	*Staphylococcus*	0.000272	0.2120	rare	fast
*S14*	Oxalobacteraceae		0.000000	0.1778	rare	fast
*S15*	Enterobacteriaceae	*Pantoea*	0.000340	0.1577	rare	fast
*S16*	Pseudomonadaceae	*Pseudomonas*	0.000000	0.1625	rare	fast
*S17*	Propionibacteriaceae	*Microlunatus*	0.000000	0.1245	rare	fast
*S18*	Xanthomonadaceae	*Pseudoxanthomonas*	0.000000	0.1253	rare	fast
*S19*	Staphylococcaceae	*Staphylococcus*	0.004340	0.1834	common	fast
*S20*	Nocardioidaceae		0.002830	0.1614	common	fast
*S21*	Bacillaceae	*Bacillus*	0.011025	0.1438	common	fast
*S22*	Intrasporangiaceae	*Terracoccus*	0.069526	0.1277	common	fast
*S23*	Pseudomonadaceae	*Pseudomonas*	0.087731	0.1860	common	fast
*S24*	Paenibacillaceae	*Paenibacillus*	0.015587	0.1144	common	fast

## Results

There was a significant effect of taxon category (*Rare/Slow*, *Common/Slow, Rare/Fast* and *Common/Fast)* on the relative abundance of bacterial taxa in the constructed communities that interacted with nutrient and predator treatment (Table 2). Since the three‐way interaction was due to only one group with a statistically significant interaction between the treatments, the main effects are shown first, followed by the interactive effects (Supporting Information Fig. S2).

**Table 2:** Statistical results of linear mixed effects model for the change in relative abundance dependent in nutrient level, predator and taxon category and for the difference in relative abundance with nutrient level or predator between every two taxon categories.

Model	Dependent variable	Explanatory variables	Factor	*F*	*p*
Linear mixed model	Relative abundance	Nutrients, Predator, Taxon category	Nutrients	0.01_3,3718_	1
			Predator	0.1_1,3718_	0.75
			Taxon category	2.1_3,19_	0.13
			Nutrient:Taxon category	7.9_9,3718_	<0.01*
			Predator: Taxon category	11.3_3.3718_	<0.01*
			Predator:Nutrient	0.02_3,3718_	1
			Predator:Nutrient: Taxon category	2.0_9,3718_	0.04*
Linear mixed model	Relative abundance	Nutrients, Taxon category	Category Common/Fast: Common/Slow	1.0_1,1806_	0.32
			Category Common/Fast: Rare/Fast	17.2_1,1870_	<0.01*
			Category Common/Fast: Rare/Slow	0.7_1,2066_	0.41
			Category Common/Slow: Rare/Slow	2.9_1,1870_	0.09
			Category Common/Slow: Rare/Fast	18.7_1,1675_	<0.01*
			Category Rare/Fast: Rare/Slow	13.4_1,1936_	<0.01*
Linear mixed model	Relative abundance	Predator, Taxon category	Category Common/Fast: Common/Slow		0.37
			Category Common/Fast: Rare/Fast	9.8_1,1871_	<0.01*
			Category Common/Fast: Rare/Slow	6.5_1,2064_	0.01*
			Category Common/Slow: Rare/Slow	3.0_1,1868_	0.08
			Category Common/Slow: Rare/Fast	10.9_1,1675_	<0.01*
			Category Rare/Fast: Rare/Slow	20.5_1,1933_	<0.01*

* indicates significant effects with a *p*‐value <0.05.

On average, individual taxa belonging to the *Rare/Slow*, *Common/Slow* and *Common/Fast* category had low relative abundances of 4% (*SE* = 0.2%), 2% (*SE* = 0.02%) and 3% (*SE* = 0.02%), respectively, whereas taxa in the category of *Rare/Fast* bacteria dominated the communities with a relative abundance of 10% (*SE* = 0.5%). The categories reached average abundances of 24% (*SE* = 1%), 48% (*SE* = 2%), 9% (*SE* = 0.7%) and 19% (*SE* = 0.9%) for the *Rare/Slow*, *Rare/Fast*, *Common/Slow* and *Common/Fast* respectively. Statistically, the *Rare/Fast* category had a tendency to be more abundant than the *Common/Slow* category (*t* = −2.2_9_, *p* = 0.06). There variation between the individual taxa of this category points out that not all taxa responded the same (Table 2).

### *Effect of nutrients*

**Figure 1:**
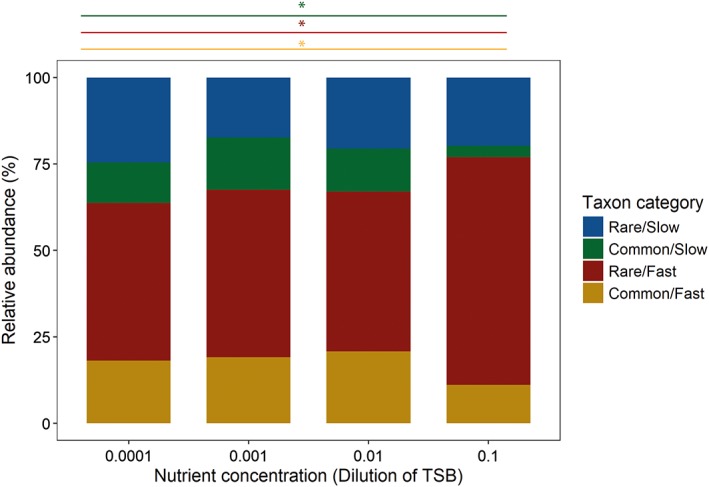
Average relative abundance of the four bacterial taxon categories at different nutrient concentrations; relative abundances are averaged over predation treatments and communities; significant linear relationships of abundance with nutrient concentration are indicated with a coloured line for the relevant taxon categories and an *.

Taxa with high potential growth rates increased significantly in abundance under increased nutrient concentration. Fast‐growing taxa increased on average by almost 50% from lowest to highest nutrient concentration (*t* = 3.4_21_, *p* < 0.01). In contrast, taxa with low potential growth rates did not respond to nutrient concentration (*t* = 1.6_21_, *p* = 0.12; Supporting Information Fig. S3). However, potential growth rate and taxon abundance in the field had an interactive effect on taxon abundance in the constructed communities. Increased nutrient concentration significantly enhanced the average relative abundance of the *Rare/Fast* category (*t* = 3.2_869_, *p* < 0.01), while it significantly decreased the relative abundance of the *Common/Fast* and *Common/Slow* groups (*Common/Fast*: *t* = −2.7_1001_, *p* < 0.01, *Common/Slow*: *t* = −4.3_*804*_, *p* < 0.02). Nutrient concentration did not influence the relative abundance of the *Rare/Slow* category (*t* = −1.2_1065_, *p* = 0.23; Fig. 1). As a consequence, increased nutrient concentration significantly promoted the *Rare/Fast* category more than the other categories (Table 2).

### *Effects of predation*

**Figure 2:**
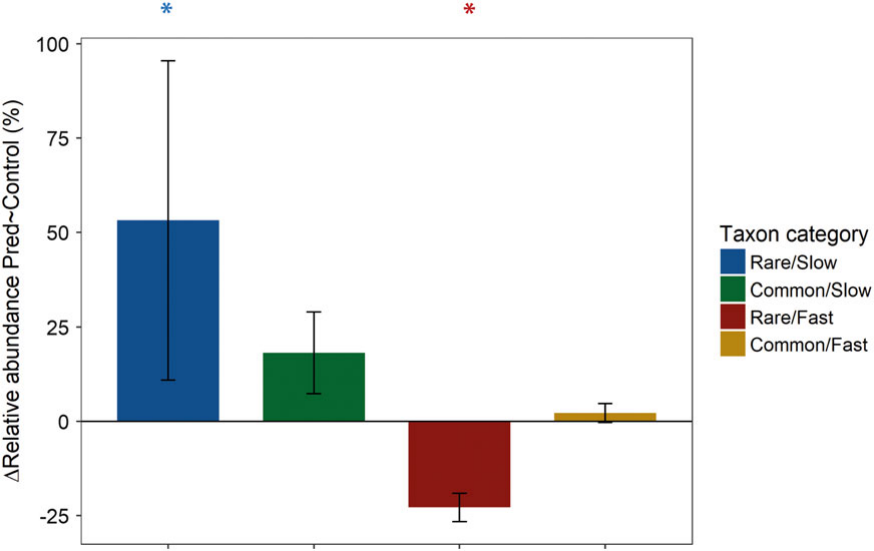
Average percentage change in relative abundance of the four bacterial taxon categories between the predation‐free control and the predation treatment; relative abundances are averaged over nutrient concentrations and communities; error bars represent the standard error; significant changes in relative abundance by predation are indicated by *.

Although, predators reduced the relative abundance of several rare taxa, the overall effect of predation on rare taxa was not significant (Supporting Information Fig. S4). However, predation affected the four taxon categories differently. The *Rare/Fast* category significantly declined with predation by 20% on average (*t* = −2.8_867_, *p* < 0.01) and this decline was significantly stronger than for all other groups (Fig. 2 and Table 2). Under predation, the *Rare/Slow* category significantly increased in abundance by 49% on average (*t* = 3.9_1036_, *p* < 0.01), whereas there was only a trend for the *Common/Slow* category (*t* = 1.9_802_, *p* = 0.05). The *Rare/Slow* category showed a significantly stronger increase than the *Common/Fast* category, whereas there was only a tendency that the *Rare/Slow* category increased more strongly than the *Common/Slow* category (Table 3).

**Table 3:** Relative abundance and standard deviation of the individual taxa in the category Rare/Fast under the different treatments.

Species	Nutrient level	Predation	Relative abundance (%)	Standard deviation
S13			3.67E+00	5.83E+00
S14			1.77E−02	2.19E−02
S15			7.45E+00	6.79E+00
S16			1.32E+01	1.57E+01
S18			2.42E+01	2.20E+01
S13	1.00E−04		8.81E+00	9.37E+00
S13	0.001		2.81E+00	2.62E+00
S13	0.01		1.61E+00	1.60E+00
S13	0.1		1.45E+00	1.93E+00
S14	1.00E−04		3.91E−02	2.62E−02
S14	0.001		2.03E−02	1.61E−02
S14	0.01		5.24E−03	7.19E−03
S14	0.1		5.33E−03	1.19E−02
S15	1.00E−04		8.32E+00	7.93E+00
S15	0.001		6.46E+00	5.73E+00
S15	0.01		5.30E+00	5.87E+00
S15	0.1		9.66E+00	6.75E+00
S16	1.00E−04		2.88E+00	3.73E+00
S16	0.001		8.82E+00	9.99E+00
S16	0.01		9.76E+00	1.02E+01
S16	0.1		3.09E+01	1.77E+01
S18	1.00E−04		2.23E+01	1.94E+01
S18	0.001		2.73E+01	2.43E+01
S18	0.01		2.64E+01	2.47E+01
S18	0.1		2.07E+01	1.91E+01
S13		no	3.74E+00	5.40E+00
S13		yes	3.60E+00	6.25E+00
S14		no	1.80E−02	2.36E−02
S14		yes	1.74E−02	2.06E−02
S15		no	8.97E+00	7.65E+00
S15		yes	5.97E+00	5.48E+00
S16		no	1.31E+01	1.56E+01
S16		yes	1.33E+01	1.59E+01
S18		no	2.88E+01	2.27E+01
S18		yes	1.96E+01	2.04E+01

Relative abundances are averaged over communities.

**Figure 3:**
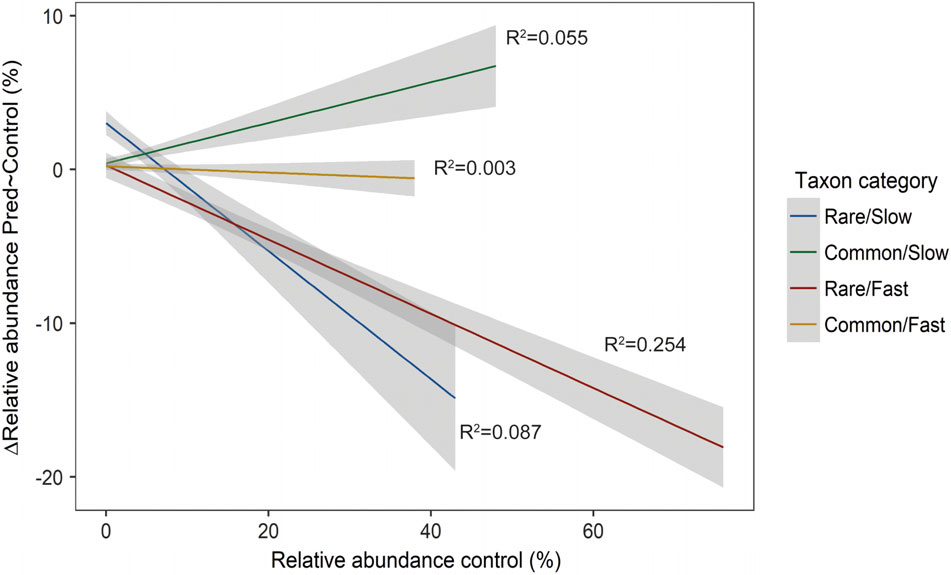
Relationship between the relative abundance in the predator free control (*X*‐axis) and the reduction of relative abundance with the addition of predators (*Y* axis) for bacterial traits belonging to the four different bacterial taxon categories. This relationship can be used as an indication of a trade‐off between competitiveness and resistance to predation; shown is a linear model fit with standard error.

Most taxa showed a negative relationship between relative abundance in the control and loss by predation (Supporting Information Fig. S5 and Table S3). However, taxon categories differed with respect to the magnitude of this response. No significant effect of category was detected when testing for frequency‐dependent predation effects. Averaged over all nutrient levels *Rare/Fast* and *Rare/Slow* taxa showed a significantly negative relationship between relative abundance in the control and loss by predation (*Rare/Fast*: *t* = −12.0_1,420_, *p* < 0.01, *Rare/Slow*: *t* = −7.0_1,509_, *p* < 0.01). In turn, this relationship was significantly positive for *Common/Slow* taxa (*t* = 4.6_1,367_, *p* < 0.01) and there was no significant relationship for the *Common/Fast* category (*t* = −1.2_1,479_, *p* = 0.21; Fig. 3 and Supporting Information Fig. S8). The relationship between relative abundance in the control and reduction by predation differed significantly between taxon categories (*F* = 62.8_3_, *p* < 0.01) except for *Common/Fast* and *Rare/Slow* (Supporting Information Table S4).

### *Effect of nutrient concentration on predation*

**Figure 4:**
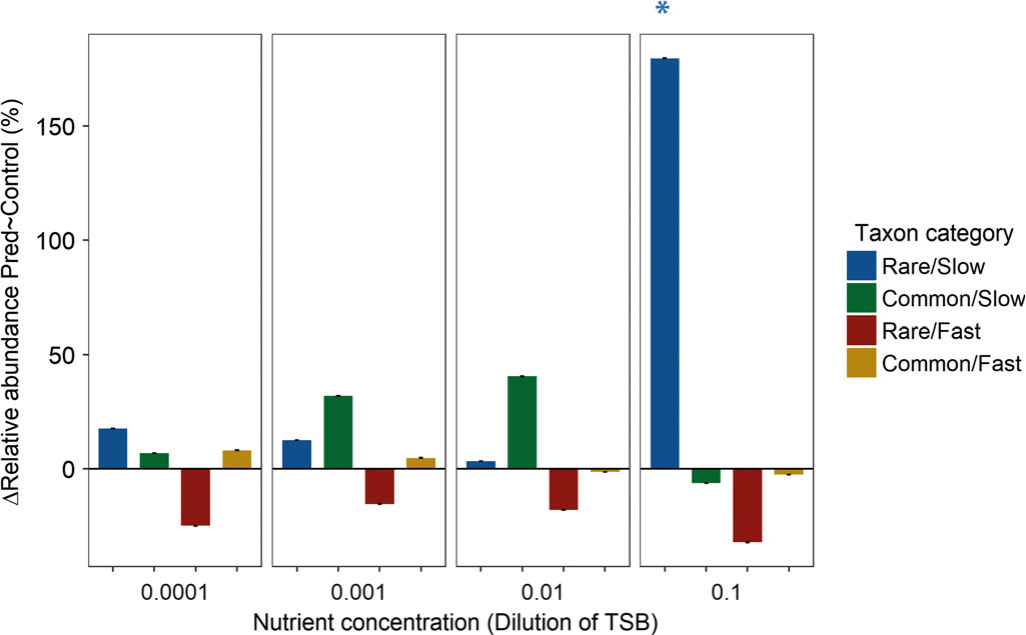
Average percentage change difference in relative abundance between the control and the predation treatment of the four bacterial taxon categories at different nutrient concentrations; results are averaged over communities; error bars represent the standard error; significant changes in relative abundance by predation are indicated by *.

There was no interactive effect of nutrient concentration and predation on the abundance of taxa that were rare or abundant in the field (*F*_3,3732_ = 0.1, *p* = 0.95, Supporting Information Fig. S6). However, the relative abundance of the *Rare/Slow* category depended on both the effects of nutrient concentration and predation: relative abundance increased significantly in the presence of predators and at the highest nutrient concentration (Fig. 4 and Supporting Information Table S5). The differences between categories were significant despite high coefficients of variation within the four groups (CV, *Rare/Slow*: 1.5, *Common/Slow*: 2.0, *Rare/Fast*: 1.2, *Common/Fast*: 1.5; for single taxa responses and statistics see Supporting Information Methods; Table S6 and Fig. S7).

**Figure 5:**
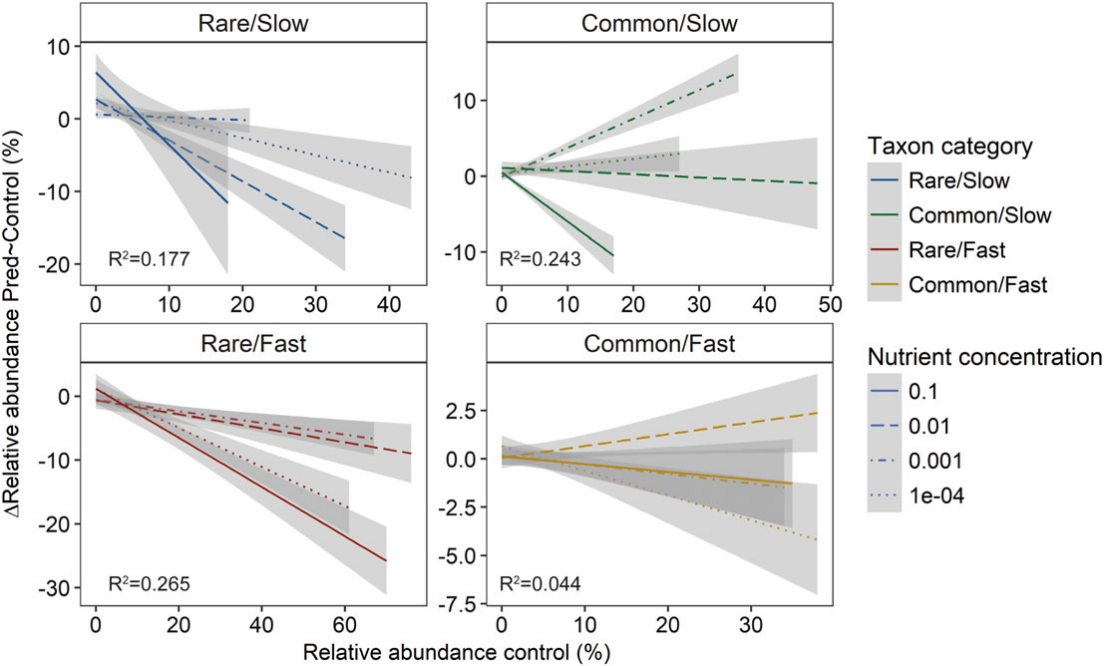
Relationship between the relative abundance in the predator free control and the reduction of relative abundance with the addition of predators at four different nutrient concentrations (indicated by different line types) and for the four taxon categories in the four panels as indication of a trade‐off between competitiveness and resistance to predation; shown is a linear model fit with standard error. *R* ^2^ values are given for each taxon category.

Nutrient concentration had a significant effect on the relationship between relative abundance in the control and loss from predation for the *Rare/Slow*, *Common/Slow* and *Common/Fast* taxon categories. For *Rare/Slow* and *Common/Slow* taxon categories, the relationship became more negative with increasing nutrient concentration, whereas it became more positive for the *Common/Fast* taxon category at a concentration of 0.01‐strength TSB (Fig. 5 and Supporting Information Fig. S10 and Table S5). Consequently, the difference between the *Rare/Slow* and *Common/Fast* categories was most pronounced at 0.01‐strength TSB. Finally, the difference between the *Common/Slow* and *Common/Fast* taxa was only significant at high nutrient levels (Supporting Information Table S7).

## Discussion

In this study, we tested the individual and combined effects of predation and the presence of competitors on the relative abundances of bacterial taxa in experimentally manipulated communities under different nutrient concentrations. We used cultivated bacteria that were rare or common in the field with either high or low potential growth rates. Rarity and commonness in this study were defined as relatively low or high local abundance, respectively, based on the average relative abundance of 16S rRNA gene sequences in a sequencing database from the same soil. We expected individual effects of predation and the presence of competitors on taxon abundances and, in addition, a trade‐off between competitive success and sensitivity to predation in constructed communities. Our results show that taxa, which become abundant without predators, are on average more negatively affected by the presence of predators in the constructed communities. This result points to a potential trade‐off between competitiveness and predation resistance. However, the taxa examined varied considerably in abundance, as well as in their responses to the experimental factors. In the following, we first discuss the individual effects of predation and competition for nutrients, and then the combined effects.

According to our first hypothesis, we expected taxa with high potential growth rates to increase in abundance with increasing nutrient concentration. However, most taxa that were common in the field and that had been characterized as fast‐growing *in vitro,* declined in abundance with increasing nutrient concentration, similar to the slow‐growing taxa. This is in contrast to studies showing a positive relationship between growth rate and competitive ability in nutrient‐rich environments (Gottschal, 1985; Fierer *et al*., 2007). Since the abundant taxa previously have shown the ability to grow fast under high nutrient concentrations (Kurm *et al*., 2017), our finding suggests that these bacteria are inferior competitors despite their high potential growth rates. This might be explained by traits other than rapid growth and nutrient uptake (i.e. exploitative competition), which influence their abundance in communities. For example, competition by the production of antimicrobial compounds (i.e. interference competition) and other such mechanisms might play a role as well (Hibbing *et al*., 2010). Bacteria of the genus *Pseudomonas* often show the capability of producing several inhibiting compounds (Haas and Defago, 2005). Nevertheless, it should be noted that in the present study no bacterial taxa with a relative abundance of 1% or more could be included. Taxa that were more abundant might have caused stronger competition. In addition, traits that determine how bacteria react to abiotic factors, such as moisture, can affect their abundance in both soil and the constructed microcosms (Lennon *et al*., 2012). A thorough investigation of the traits of individual taxa or their estimation by whole genome sequencing might enable us to more accurately predict taxon abundance in different environments.

We also tested the hypothesis that taxa that are rare in the field would be most negatively affected by protist predation in the constructed communities. While overall predation by protists reduced abundances of the taxa that were dominant in the control, the effect was most pronounced for taxa in the *Rare/Fast* category. Although, this did not change the rank order of the four taxon categories, a higher predator density than used in the present study might lead to a lower abundance of these taxa. Moreover, in soil, predation pressure on bacteria is likely to be stronger because of the huge taxonomic and functional diversity of protist predators and nematodes (Geisen *et al*., 2018). Protist species select their prey with feeding preferences that differ depending on the protist predator and the bacterial taxon upon which they prey (Schulz‐Bohm *et al*., 2016). Therefore, a higher predator diversity may reduce the strength of competition for nutrients and increase bacterial diversity (Saleem *et al*., 2012). We found a negative relationship between abundance in the absence and presence of predation for both rare, but not for the abundant, taxon categories. This negative effect of predation is only partly due to preferential feeding of the predators on the most abundant taxa, as it persisted regardless of the abundance of the taxa in the constructed communities.

Our results suggest a trade‐off between competitiveness and predation resistance, because taxa with high potential growth rates and low abundance in the field were more negatively affected by predation. This is in support of the kill‐the‐winner hypothesis, as taxa that can grow fast may be less resistant to predation or viral lysis (Pernthaler, 2005; Thingstad *et al*., 2014). This relationship is supposed to be due to the fitness costs of predation resistance. For example, for phytoplankton morphological defences, such as bulky shapes, small cell sizes, colony formation and toxin production may lead to a reduced growth rates and, therefore, reduced competitiveness (Pančić and Kiørboe, 2018). Our study shows that not all fast‐growing taxa were equally negatively affected, which might be due to different growth‐defence trade‐offs. These differences can arise from contrasting mechanisms of resistance (Friman *et al*., 2013) or from specific mutations underlying the mechanisms, as has been observed for bacterial resistance to phages (Jessup and Bohannan, 2008). Taxa that are rare in the field might have higher costs of anti‐predator defences. If so, this will reduce their competitive ability and, consequently, their fitness. However, we did not specifically test for resistance mechanisms of the different taxa, so that further studies would be required to elucidate the mode of operation.

Our results are partly supporting our third hypothesis that predation and nutrient concentration interact to affect taxon abundance. Slow‐growing taxa showed a more negative relationship between abundance with and without predation at higher nutrient levels. This is in accordance with studies finding predation to be more important than competition for nutrients at high compared to low nutrient concentrations (Bohannan and Lenski, 2000b; Corno and Jürgens, 2008). Although, this was not more than a marginal trend for the Rare/Slow group, we conclude that other factors than nutrient condition will have affected abundance. In contrast to our hypothesis, the *Common/Fast* category was only weakly affected by predation at all nutrient concentrations. Toxin and volatile production by taxa belonging to the genera *Pseudomonas*, *Paenibacillus* and *Bacillus* might have enhanced their resistance to predation (Jousset *et al*., 2009; Schulz‐Bohm *et al*., 2016). However, these interactions will be highly taxon‐ and compound‐specific (Schulz‐Bohm *et al*., 2016), and measuring such interactions was beyond the scope of the present study. Also for the *Rare/Fast* group, the consistent negative relationship between competitiveness and predation resistance was unaffected by nutrient availability. In addition, there was no interaction between the nutrient concentration and predation treatments for the taxon categories except for the taxon S4 from the Rare/Slow category. This finding indicates that not relative abundance is affected by predation and nutrient concentration, but rather the trade‐off between competitive ability in the absence of predators and predation resistance.

Most surprisingly, bacterial taxa belonging to the rare fast‐growing (*Rare/Fast)* category dominated all communities, in spite of their low abundances in the field. That might be partly due to technical issues, such as classification according to a sequencing database in which OTUs might be composed of several taxa. Nevertheless, the classification of different taxa into one OTU does not negatively influence the classification of the rare taxa used in the present study, as there was only minor variability in relative abundance of the rare taxa in the sequencing database (Supporting Information Fig. S12). Primer biases might lead to an underestimation of the abundance of certain taxa (v. Wintzingerode *et al*., 1997), but this is unlikely to cause the present results since strains classified as ‘rare’ or ‘common’ were not phylogenetically clustered and all primers perfectly matched the sequences of the strains used (except for taxon S17 which was not detected in the constructed communities). Still, there was considerable variation within the Rare/Fast category. Two taxa became highly abundant in the constructed communities, whereas two were of moderate abundance and one stayed rare. This variability might have led to an extraproportional effect of the highly abundant taxa. However, the high proportion of taxa in the Rare/Fast category that was relatively abundant in predator‐free communities and decreased by predation indicates that some taxa might become common when not inhibited by predation. Some taxa were also affected by nutrient concentration or stayed low abundant in all treatments. Together, these results indicate that low abundance can be caused by a variety of factors and that it appears to be taxon dependent.

Variation between taxa could have concealed weak effects of the different treatments. Moreover, other constraints of our experimental system might have prevented us from finding stronger patterns. For example, although, the taxon richness used here is large compared to most microbial competition studies (Bohannan and Lenski 2000a,b; Jiang and Adams Krumins 2006), our experimental communities were considerably less diverse than natural communities with respect to both bacterial prey taxa and predator species. It has been shown that more simple interactions cannot accurately predict the behaviour of complex communities (McClean *et al*., 2018). Moreover, our setup did not enable us to separate inter from intraspecific competition. The experimental system was also less heterogeneous than the soil environment. Heterogeneity is supposed to enable the coexistence of a high number of taxa that differ in their niche requirements (Torsvik *et al*., 1996; Zhou *et al*., 2002). Thus, in rather homogenous batch cultures some rare taxa might behave as abundant ones, while their competitiveness might differ under field conditions. On the other hand, the lack of a clear pattern for the behaviour of rare and abundant taxa indicates that neither competition for nutrients nor predation alone play a dominant role in controlling taxon abundance. Instead, other factors might influence taxon abundance, such as abiotic conditions (Lauber *et al*., 2009), dispersal ability (Smith *et al*., 2018) or order of arrival (Fukami, 2015). The interplay of these factors and differences in traits between the individual taxa can enable coexistence and the persistence of rare taxa in addition to competitive exclusion (Chase *et al*., 2002; Torsvik *et al*., 2002). Importantly, the present study demonstrates that causes of rarity are likely to differ between taxa, and that some rare taxa have the potential to become abundant under more favourable conditions, whereas others appear less capable of doing so.

We conclude that the *in vitro* growth rate of bacterial taxa is not a good predictor of their competitive ability and that potential growth rate or competitive ability does not sufficiently explain bacterial abundance in field soil. We show a negative relationship between competitiveness and predation resistance in soil bacteria and conclude that this relationship on average is stronger for rare than for common taxa. This inverse relationship suggests a trade‐off between competitiveness and predation resistance. Our results suggest predation to be an important factor explaining bacterial rarity under natural conditions. Future work is needed to test if the correlation that we observed is based on a mechanistic trade‐off in resource investment at the expense of predation resistance.

## Experimental procedures

### *Bacterial isolates*

All bacterial isolates used in this study originate from a single cell or colony (for additional information on the cultivation approach, see Supporting Information Methods). We can assume that all isolates represent single bacterial strains, since the isolates originating from a single colony (S4 and S6) showed no sequence variation and clear chromatograms without ambiguous base positions following the Sanger sequencing technique. We defined rarity as a ‘low local abundance’, which was determined by matching isolate sequences to a sequencing database prepared from soil samples obtained from the location of the isolates' origin. Relative abundance was calculated as the relative abundance of 16S rRNA gene sequences of the matching OTU (see Supporting Information Methods). The relative abundances used in this study represented an average of seven soil samples. We classified the bacterial isolates into rare and common taxa. Taxa with a relative abundance of < 0.01% of all obtained reads were classified as rare and taxa with a relative abundance > 0.01% were classified as abundant in the field respectively (for relative abundances in the field soil of all taxa see Table 1). This particular cut‐off was chosen as a rather conservative value when defining rare bacterial taxa, and has previously been employed by Galand and colleagues (2009). We further classified the isolates into ‘slow’‐ and ‘fast’‐growing based on their average potential growth rate in tryptone soy broth (TSB; Table 1). Taxa with a growth rate < 0.1 h^−1^ were considered to be slow‐growing, whereas taxa with a growth rate > 0.1 h^−1^ were considered as fast‐growing. This differentiation was based on the range and distribution of growth rates in our isolate collection (average growth rate: 0.098 h^−1^, min: 0.007, max: 0.211, for distribution see Supporting Information Fig. S1). Hence, the 24 taxa were grouped into four categories of six taxa each: (a) rare and slow‐growing (*Rare/Slow*), (b) common and slow‐growing (*Common/Slow*), (c) rare and fast‐growing (*Rare/Fast*) and (d) common and fast‐growing (*Common/Fast*) taxa (Table 1). This approach enabled us to detect potential differences between taxa with different growth rates that were rare or common in the field respectively.

### *Protist predators*

We used three protist isolates that were all member of the amoeboid genus *Vannella* sp. (supergroup: Amoebozoa, family: Vannellidae) isolated from grassland soil at the ex‐arable land chronosequence De Mossel site described in Morriën and colleagues (2017) that is close to the site of the origin of the bacterial isolates. For cultivation of protists see Supporting Information Methods.

### *Community design and microcosm construction*

We designed 24 bacterial communities that contained 12 taxa each, with three taxa from each of the four abundance/growth rate categories, resulting in the taxon categories *Rare/Slow, Common/Slow*, *Rare/Fast* and *Common/Fast*. For each group, the three species were drawn from the pool of six taxa in a constrained random approach avoiding overrepresentation of particular taxa or taxon combinations (Supporting Information Table S2). Microcosms consisted of Nunc 1.0 ml 96‐ Deep‐well plates (Thermo Fisher Scientific, Waltham, MA, USA) containing 850 μl of medium (see below) and 50 μl MgSO_4_‐ buffer. We inoculated every community into each of four concentrations of liquid TSB (0.1, 0.01, 0.001 and 0.0001 TSB) resulting in 96 microcosms. All 24 communities at all nutrient concentrations were setup four times, crossed in a full‐factorial design with each of the three protist predators and a predator‐free control, resulting in 384 microcosms in total (24 communities × 4 nutrient concentrations × [3 predators +1 control]). We inoculated one additional replicate of each community in 0.1 TSB and harvested them immediately to serve as a baseline control to detect potential sequencing biases.

Communities were constructed with a pipetting robot (Freedom Evo, Tecan, Männedorf, Switzerland), which transferred 8.3 μl of each bacterial monoculture into the respective well to create the designed communities consisting of 50 μl mixed bacterial culture (yielding a final concentration of 3 × 10^4^ cells.ml^−1^), 50 μl protist culture in MgSO_4_‐ buffer (with a cell density of 5 × 10^4^ cells.ml^−1^ yielding a final concentration of 2500 cells.ml^−1^) or 50 μl MgSO_4_‐ buffer for the control treatment leading to a final volume of 1 ml per well. These high concentrations of predators were applied to compensate for the low predator diversity compared to field soil. We covered the plates with breathable rayon sealing film (VWR, Radnor, PA, USA) and incubated them for 7 days at 25°C. Starting 24 h after community construction the plates were agitated gently at 100 r.p.m. until harvest to prevent anaerobic conditions. At harvest the plates were closed with a lid and were stored at −20°C until DNA extraction.

### *DNA extraction and Illumina MiSeq library preparation*

We extracted DNA from all microcosm communities using the QIAmp DNA Mini kit (Qiagen, Venlo, The Netherlands) with a pre‐treatment with lysozyme and proteinase K according to manufacturer's instructions. Subsequently, we amplified the V4‐region of the 16S rRNA gene using custom primers (Supporting Information Table S3). The reverse primer was barcoded with a 12 bp goaly barcode enabling multiplexing. Each sample was amplified in triplicate. All PCR reactions contained 11.75 μl MQ‐water, 10 μl 5 Prime Hot Mastermix (Quantabio, Beverly, MA, USA), 1.25 μl BSA, 0.5 μl of forward and reverse primer (10 μM final concentration) and 1.0 μl genomic DNA. The PCR conditions were as follows: an initial denaturation step of 94°C for 5 min, 35 cycles of 45 s at 94°C, 60 s at 50°C and 90 s at 72°C, followed by a final extension step for 10 min at 72°C.

We purified the PCR products using Agencourt AMPure beads (Beckman Coulter, Indianapolis, IN, USA) using a ratio of 1:0.7 of PCR product to bead volume. The purification was carried out according to the manufacturer's protocol and purified products were diluted in 30 μl MQ‐water. We then measured the concentrations of the purified PCR products with a fragment analyser (Advanced Analytical, Ankeny, IA, USA) using the standard sensitivity NGS fragment analysis kit (Advanced Analytical). The products were mixed in equal nanogram quantities and sent to BGI (Shenzhen, China) for 150 bp paired‐end sequencing with Illumina MiSeq. The three custom sequencing primers included two primers for reading the amplicon from each side and one for reading the barcode [for primer sequences see (Apprill *et al*., 2015)].

### *Sequence analysis*

We merged paired end reads using the fastq‐mergefiles option implemented in VSEARCH version 1.0.10 (Rognes *et al*., 2016), converted all sequences to the FASTA format and concatenated them to a single file. We clustered sequences into OTUs by de‐replication with the UPARSE strategy using the UCLUST smallmem algorithm (Edgar, 2010) and removed chimeric sequences with the UCHIME algorithm (Edgar *et al*., 2011). To match the sequences to the original 24 taxa, we created a custom sequence database consisting of Sanger sequences from all isolates from the same region as the Illumina reads. In addition, we obtained neighbouring sequences of 97%–100% identity for each isolate from the SILVA‐database and NCBI‐GenBank and included those in the custom database. Subsequently we mapped all reads before the de‐replication step to the custom database with the usearch_global method from VSEARCH and a sequence identity of 97%. However, for the taxa S9 and S20 (Nocardioidaceae), S13 and S19 (Staphylococcaceae) and S16 and S23 (Pseudomonadaceae), mapping was done at a 99% identity because of the high similarity between the isolate sequences. Most (97%) sequences were successfully mapped to one of the reference sequences and we summed all sequences for each of the 24 species per sample. We removed samples that contained less than 1000 sequences from the dataset. Relative abundance of each taxon in each sample was calculated, followed by normalization with the 16S‐copy number from the nearest neighbour sequence in the database by Větrovský and Baldrian (2013).

Most communities differed in taxon composition, and the number of taxa differed from the original species design, as well. Even the 24 communities that were frozen immediately after construction by the pipetting robot showed a different composition than intended. Taxon S17 was not detected in the entire sequencing dataset. The absence of taxon S17 might be explained by a mismatch between the forward primer and the 16S rRNA gene. The deviations from the designed communities might result from sequencing bias and/or cross‐contamination. While this clearly is undesired ‘noise’ in our dataset, the majority of the communities consisted of the intended taxa, so we kept all retrieved sequences in the analysis and analysed the relative abundances of the taxa in relation to the treatments and the designated taxon categories.

### *Statistical analysis*

All statistical analyses were performed in R version 3.4.0 with R Studio (R Core Team, 2016). A linear mixed effect model testing the effects of the three different predators on taxon abundance with nutrient concentration and community as random factors indicated no difference between the three protist predators, except for taxon S18, for which the effects of two of the three predators on abundance differed significantly (*t* ratio: −3.3, *p* = 0.01). Therefore, given the similar response of all but one of the bacterial taxa, we averaged the relative abundance over the three different predators, resulting in one predation treatment.

We analysed whether relative abundance in the constructed communities was dependent on nutrient concentration, predation and taxon category (i.e. *Rare/Slow, Common/Slow, Rare/Fast* and *Common/Fast* respectively) by fitting a linear mixed model from the lme4‐package with taxon as the random factor and obtaining the minimal model using the step() function from the lmerTest package (Bates *et al*., 2015). The same model was fitted for each taxon separately to test how many taxa in each group followed the main group effect. Moreover, we used linear mixed effect models to assess if the differences in relative abundances between the groups changed with nutrient concentration. This was done by fitting linear mixed effect models for each group pair with predation and taxon as a random factor. We performed a similar test for differences in the slope between control and predator treatment between the groups with nutrient level and species as random factors to assess the predator effect only.

For each taxon, we tested the relationship between relative abundance and abundance change in the presence of predation with a Spearman correlation between relative abundance in the control (which is a proxy for competitiveness) and change in relative abundance with predation (which is a proxy for predation resistance). Using a Bonferroni‐correction did not change the results; therefore, we report the uncorrected values. To test for a potential relationship between competitiveness and predation resistance independent of taxon abundance, a linear mixed model was fit with relative abundance in the control as the response variable and the effect size of the response to predation as the explanatory variable. We used taxon category and nutrient concentration with community nested in nutrient concentration as a random factor. As the random effects were not significant, we fitted simple linear models for each pair of taxon categories. In addition, a separate model was fitted to test for the effect of nutrients, with nutrient concentration included as a fixed factor. Furthermore, we fitted the same model for each taxon category and each group pair separately to determine changes within and between groups with nutrient level.

## Supporting information


**Appendix 1:** Supplementary Information.

**Appendix 2:** Supporting Information.
