# Supplementary material for: Competition and predation as possible causes of bacterial rarity
Source: Environ Microbiol. 2019 Mar 18;21(4):1356–68. doi: 10.1111/1462-2920.14569 (PMC6850713; doi:10.1111/1462-2920.14569)
Supplement: Supplementary file 1 — Appendix 1: Supplementary Information. [file EMI-21-1356-s001.docx]

**Supplementary Information**

This supplementary includes methods detailing cultivation of the microbial organisms used in this study and results of an analysis after omitting a part of the dataset. Tables give details on the microbial isolates and the construction of communities as well as statistical results. Figures illustrate results from the main text.

Supplementary methods

*Cultivation of bacterial isolates*

We chose 24 bacterial taxa from a collection of taxa isolated from a soil of a long-term biodiversity experiment on ex-arable land near Ede (Gelderland, The Netherlands) (for details on the isolation procedure see Kurm, van der Putten et al. (2017)). In brief, we used both flow cell sorting and a streak plating method in combination with a range of different oligotrophic growth media for bacterial cultivation. In the flow cell sorting approach, individual bacterial cells with a size <0.5 μm were sorted from a soil sample into single wells of 96-well plates containing 6 different media (tryptone soil agar at a 1/10 and 1/100 dilution, water yeast agar, dilute nutrient broth agar and soil agar with and without the addition of nutrients). For the streak plating approach 200 μl of a 10^-7^ diluted soil solution were spread over petri-dishes containing 2 different media (soil extract medium with salts and VL55 medium + xylan). All plates were checked for visible bacterial growth every other day. The partial 16S rRNA genes of the isolated bacterial taxa were sequenced by Sanger sequencing to determine taxonomic placements of the bacterial strains. For determination of their abundance in field soil the sequences were compared to a 454-sequencing database of the v4-region of a microbial community from the same soil and matched to the closest related OTU. The relative abundance of sequences clustered into the respective OTU were subsequently taken as the relative abundance of the taxa in soil. We measured potential growth rates of the isolated taxa in tryptone soy broth (TSB; 3 g l^-1^ tryptone soy broth; Table S1) (Kurm, van der Putten et al. 2017).

Prior to community construction all bacterial taxa were pre-cultured separately for 5 days in liquid TSB at 25°C and 180 rpm agitation on a flatbed shaker. One day prior to the set-up we washed the cultures 3x in sterile MgSO_4_ buffer (2.46 g.l^-1^ MgSO_4_). Cell densities were determined by staining subsamples of each bacterial solution with SYBR green at a final concentration of 1:10 000 (Sigma-Aldrich, St. Louis, Missouri, USA) for minimally 15 min counting by flow cytometry in an Accuri C6 Sampler (BD Biosciences, Franklin Lakes, New Jersey, USA). We adjusted the separate solutions to an equal density of 6x10^5^ cells.ml^-1^ in MgSO_4_-buffer and stored them at 4°C for maximally 24 h until community construction.

*Protist cultivation*

We prepared precultures in WG-medium (Geisen, Weinert et al. 2014) with *E. coli* as a food source. Two days prior to community construction, we treated the cultures with three different antibiotic solutions to supress growth of *E. coli* using 25 μl 100 mg.ml^-1^ ampicillin, 10 μl 20 mg.ml^-1^ rifampicin and 5 μl 50 mg.ml^-1^ kanamycin per 500 μl of protist culture. One day prior to community construction, we washed protist cultures 3x in sterile MgSO_4_ buffer, counted them microscopically and adjusted them to a density of 50000 cells.ml^-1^. Subsequently, we stored the protist cultures at 4°C for maximally 24h until community construction.

*Protist quantification*

During harvest 100 µl from each microcosms were immediately homogenized with a fixative solution (0.1 g.ml^-1^ paraformaldehyde, 10% glutaraldehyde) at a concentration of fixative of 1:100. As the fixative caused lysis of the protist cells, only lysed cells were detected by microscopy and consequently protist cells could not be quantified reliably.

Supplementary results

*Relationship between competitive success and predation resistance*

To explore whether the results for the *Rare/Slow* category were mainly driven by the strongly responding taxon S4 we conducted an additional analysis after omitting S4 from the dataset. Here the *Rare/Slow* category still differed significantly from both the *Common/Slow* and *Common/Fast* category, but no longer from the *Rare/Fast* category (Fig. S9, Table S13).

*Effect of nutrients on the relationship between competitive success and predation resistance*

When omitting S4 from the dataset the relationship between relative abundance in the control and reduction in the predation treatment did not change anymore with nutrient level for the *Rare/Slow* category (Figure S11, Table S14).

| Table S1: Composition of the 24 artificial communities (C). 0 designates absence and 1 designates presence of a taxon in the respective community. Different colours indicate the four different taxon categories (Rare/Slow, Common/Slow, Rare/Fast, and Common/Fast). | | | | | | | | | | | | | | | | | | | | | | | | | | | |
| --- | --- | --- | --- | --- | --- | --- | --- | --- | --- | --- | --- | --- | --- | --- | --- | --- | --- | --- | --- | --- | --- | --- | --- | --- | --- | --- | --- |
| Com | Species | | | | | | | | | | | | | | | | | | | | | | | | | | |
|  | **Rare/Slow** | | | | | | **Abundant/Slow** | | | | | | | **Rare/Fast** | | | | | | | **Abundant/Fast** | | | | | | |
|  | **S1** | **S2** | **S3** | **S4** | **S5** | **S6** | | **S7** | **S8** | **S9** | **S10** | **S11** | **S12** | | **S13** | **S14** | **S15** | **S16** | **S17** | **S18** | | **S19** | **S20** | **S21** | **S22** | **S23** | **S24** |
| 1 | 1 | 1 | 1 | 0 | 0 | 0 | | 0 | 0 | 0 | 1 | 1 | 1 | | 1 | 0 | 0 | 0 | 1 | 1 | | 1 | 0 | 1 | 1 | 0 | 0 |
| 2 | 1 | 0 | 1 | 0 | 1 | 0 | | 0 | 1 | 0 | 1 | 0 | 1 | | 0 | 1 | 0 | 1 | 0 | 1 | | 1 | 1 | 0 | 1 | 0 | 0 |
| 3 | 0 | 1 | 1 | 0 | 0 | 1 | | 0 | 0 | 0 | 1 | 1 | 1 | | 0 | 1 | 1 | 1 | 0 | 0 | | 0 | 0 | 0 | 1 | 1 | 1 |
| 4 | 1 | 1 | 0 | 0 | 1 | 0 | | 1 | 1 | 0 | 0 | 1 | 0 | | 1 | 1 | 0 | 0 | 1 | 0 | | 0 | 1 | 0 | 1 | 0 | 1 |
| 5 | 0 | 1 | 0 | 1 | 1 | 0 | | 1 | 1 | 0 | 0 | 0 | 0 | | 1 | 1 | 0 | 1 | 1 | 0 | | 0 | 0 | 1 | 1 | 1 | 0 |
| 6 | 1 | 0 | 0 | 1 | 1 | 0 | | 0 | 1 | 1 | 0 | 1 | 0 | | 0 | 1 | 0 | 0 | 1 | 1 | | 0 | 1 | 0 | 1 | 0 | 1 |
| 7 | 0 | 0 | 1 | 0 | 1 | 1 | | 0 | 1 | 1 | 0 | 0 | 1 | | 1 | 0 | 0 | 1 | 0 | 1 | | 1 | 1 | 1 | 0 | 0 | 0 |
| 8 | 0 | 0 | 1 | 0 | 1 | 1 | | 0 | 0 | 1 | 0 | 1 | 1 | | 1 | 1 | 0 | 0 | 0 | 1 | | 1 | 0 | 1 | 0 | 1 | 0 |
| 9 | 1 | 0 | 1 | 1 | 0 | 0 | | 1 | 1 | 0 | 1 | 0 | 0 | | 1 | 0 | 1 | 0 | 0 | 1 | | 0 | 0 | 1 | 1 | 0 | 1 |
| 10 | 1 | 0 | 0 | 1 | 0 | 1 | | 0 | 1 | 1 | 0 | 1 | 0 | | 1 | 1 | 1 | 0 | 0 | 0 | | 1 | 0 | 1 | 0 | 0 | 1 |
| 11 | 1 | 1 | 0 | 0 | 0 | 1 | | 1 | 0 | 1 | 1 | 0 | 0 | | 0 | 0 | 1 | 1 | 1 | 0 | | 0 | 1 | 0 | 0 | 1 | 1 |
| 12 | 1 | 1 | 0 | 1 | 0 | 0 | | 1 | 1 | 0 | 0 | 0 | 1 | | 1 | 0 | 1 | 0 | 0 | 1 | | 1 | 1 | 0 | 0 | 0 | 1 |
| 13 | 0 | 0 | 1 | 1 | 1 | 0 | | 0 | 1 | 0 | 1 | 1 | 0 | | 1 | 0 | 1 | 1 | 0 | 0 | | 1 | 0 | 0 | 1 | 1 | 0 |
| 14 | 0 | 1 | 0 | 1 | 1 | 0 | | 0 | 0 | 1 | 1 | 1 | 0 | | 0 | 1 | 1 | 0 | 0 | 1 | | 0 | 1 | 1 | 1 | 0 | 0 |
| 15 | 0 | 1 | 0 | 1 | 0 | 1 | | 1 | 0 | 0 | 0 | 1 | 1 | | 1 | 0 | 0 | 0 | 1 | 1 | | 1 | 0 | 1 | 0 | 0 | 1 |
| 16 | 0 | 0 | 0 | 1 | 1 | 1 | | 1 | 0 | 1 | 0 | 1 | 0 | | 0 | 1 | 1 | 0 | 1 | 0 | | 0 | 1 | 1 | 0 | 1 | 0 |
| 17 | 1 | 1 | 0 | 0 | 0 | 1 | | 0 | 0 | 1 | 1 | 0 | 0 | | 1 | 0 | 0 | 1 | 1 | 1 | | 1 | 0 | 0 | 0 | 1 | 1 |
| 18 | 1 | 0 | 0 | 1 | 0 | 1 | | 1 | 1 | 0 | 1 | 0 | 1 | | 1 | 0 | 0 | 0 | 1 | 0 | | 0 | 0 | 1 | 0 | 1 | 1 |
| 19 | 0 | 1 | 1 | 0 | 1 | 0 | | 1 | 0 | 0 | 1 | 1 | 0 | | 0 | 1 | 0 | 1 | 1 | 0 | | 0 | 0 | 1 | 0 | 1 | 1 |
| 20 | 0 | 0 | 1 | 1 | 1 | 0 | | 1 | 1 | 0 | 0 | 0 | 1 | | 0 | 1 | 1 | 1 | 0 | 0 | | 1 | 1 | 0 | 0 | 1 | 0 |
| 21 | 1 | 0 | 0 | 0 | 1 | 1 | | 0 | 1 | 1 | 1 | 0 | 0 | | 1 | 1 | 0 | 0 | 0 | 1 | | 1 | 0 | 0 | 1 | 1 | 0 |
| 22 | 0 | 0 | 1 | 1 | 0 | 1 | | 1 | 0 | 0 | 1 | 0 | 1 | | 0 | 0 | 0 | 1 | 1 | 1 | | 0 | 0 | 1 | 1 | 1 | 0 |
| 23 | 1 | 1 | 1 | 0 | 0 | 0 | | 0 | 0 | 1 | 1 | 0 | 1 | | 0 | 1 | 1 | 0 | 1 | 0 | | 0 | 0 | 1 | 1 | 1 | 0 |
| 24 | 0 | 1 | 1 | 1 | 0 | 0 | | 1 | 0 | 1 | 0 | 1 | 0 | | 1 | 0 | 1 | 1 | 0 | 0 | | 0 | 1 | 1 | 1 | 0 | 0 |

Table S2: Custom primers used for 16S-rRNA library generation; the 12-bp barcode is marked in bold letters.

| Primer | Sequence (5’-3’) |
| --- | --- |
| 515f | AATGATACGGCGACCACCGAGATCTACACTATGGTAATTGTGTGCCAGCMGCCGCGGTAA |
| 806r | CAAGCAGAAGACGGCATACGAGAT**TCCCTTGTCTCC**AGTCAGTCAGCCGGACTACHVGGGTWTCTAAT |

Table S3: statistical results of Spearman correlations between relative abundance in the control and relative abundance reduction in the predation treatment for each taxon; significant p-values are marked in bold (n=45-93).

| Taxon | S | rho | p |
| --- | --- | --- | --- |
| S1 | 23116 | 0.21 | 0.12 |
| S2 | 222682 | -0.66 | **0.00** |
| S3 | 196490 | -0.51 | **0.00** |
| S4 | 180480 | -0.35 | **0.00** |
| S5 | 86451 | 0.12 | 0.26 |
| S6 | 187652 | -0.40 | **0.00** |
| S7 | 28073 | -0.73 | **0.00** |
| S8 | 28811 | -0.67 | **0.00** |
| S9 | 19803 | -0.30 | **0.04** |
| S10 | 138197 | -0.82 | **0.00** |
| S11 | 60856 | -0.61 | **0.00** |
| S12 | 94656 | 0.29 | **0.00** |
| S13 | 170376 | -0.31 | **0.00** |
| S14 | 33569 | -0.52 | **0.00** |
| S15 | 222778 | -0.66 | **0.00** |
| S16 | 149394 | -0.11 | 0.27 |
| S18 | 193488 | -0.44 | **0.00** |
| S19 | 65739 | -0.44 | **0.00** |
| S20 | 145412 | -0.33 | **0.00** |
| S21 | 41974 | -0.51 | **0.00** |
| S22 | 147850 | -0.22 | **0.04** |
| S23 | 166182 | -0.24 | **0.02** |
| S24 | 184830 | -0.47 | **0.00** |

Table S4: statistical results of pairwise linear models testing differences in the relationship between relative abundance in the control and reduction in relative abundance by predation between the four taxon categories

| Groups | t | p |
| --- | --- | --- |
| Common/Fast:Common/Slow | 3.7_3,846_ | <0.01* |
| Common/Fast:Rare/Fast | -4.3_3,899_ | <0.01* |
| Common/Fast:Rare/Slow | -0.5_3,988_ | 0.64 |
| Common/Slow: Rare/Slow | -6.5_3,876_ | <0.01* |
| Common/Slow: Rare/Fast | 7.2_3,787_ | <0.01 |
| Rare/Fast: Rare/Slow | 9.8_3,929_ | <0.01* |

Table S5: Statistical results of pairwise contrasts from a linear model testing differences in the relationship between relative abundance in the control and reduction in relative abundance by predation for different nutrient concentrations within each taxon category

| Nutrient concentration | | 0.0001-  0.001 | | | 0.0001-  0.01 | | | 0.0001-  0.1 | | | 0.001-  0.01 | | | 0.001-  0.1 | | | 0.01-  0.1 | |
| --- | --- | --- | --- | --- | --- | --- | --- | --- | --- | --- | --- | --- | --- | --- | --- | --- | --- | --- |
| Category | **t** | | **p** | **t-** | | **p** | **t** | | **p** | **t** | | **p** | **t** | | **p** | **t** | | **p** |
| Rare/slow | -2.1_503_ | | 0.16 | 0.0_503_ | | 1 | -4.3_503_ | | <0.01* | 2.3_503_ | | 0.11 | -0.2_503_ | | 1 | -5.8_503_ | | <0.01* |
| Rare/Fast | 1.0_414_ | | 0.77 | -1.1_414_ | | 0.66 | -0.9_414_ | | 0.83 | -1.7_414_ | | 0.31 | -1.5_414_ | | 0.31 | 0.6_414_ | | 0.93 |
| Common/Slow | -3.5_361_ | | <0.01* | 2.4_361_ | | 0.08 | 3.6_361_ | | <0.01* | 7.8_361_ | | <0.01* | 7.3_361_ | | <0.01* | 2.2_361_ | | 0.14 |
| Common/Fast | -0.7_473_ | | 0.91 | -3.7_473_ | | <0.01* | -0.8_473_ | | 0.85 | -2.9_473_ | | 0.02* | -0.2_473_ | | 0.99 | 2.5_473_ | | 0.11 |

Table S6: Statistical results of a linear mixed effect model testing the effect of predation and nutrient concentration on relative abundance for each taxon; significant p-values are marked in bold.

| Taxon | Treatment | Df | Sum.Sq | Mean.Sq | F.value | upper.den.df | upper.p.val | lower.den.df | lower.p.val | expl.dev. |
| --- | --- | --- | --- | --- | --- | --- | --- | --- | --- | --- |
| S1 | Predator | 1 | 0.0000 | 0.0000 | 0.3 | 134 | 0.57 | 110 | 0.57 | 0.17 |
| S1 | Nutrient | 1 | 0.0001 | 0.0001 | 30.2 | 134 | **0.00** | 110 | **0.00** | 15.98 |
| S1 | Predator:Nutrient | 1 | 0.0000 | 0.0000 | 0.1 | 134 | 0.81 | 110 | 0.81 | 0.03 |
| S2 | Predator | 1 | 0.0110 | 0.0110 | 4.6 | 185 | **0.03** | 161 | **0.03** | 1.33 |
| S2 | Nutrient | 1 | 0.0531 | 0.0531 | 22.1 | 185 | **0.00** | 161 | **0.00** | 6.41 |
| S2 | Predator:Nutrient | 1 | 0.0004 | 0.0004 | 0.2 | 185 | 0.68 | 161 | 0.68 | 0.05 |
| S3 | Predator | 1 | 0.0014 | 0.0014 | 0.8 | 184 | 0.37 | 160 | 0.37 | 0.25 |
| S3 | Nutrient | 1 | 0.0003 | 0.0003 | 0.2 | 184 | 0.67 | 160 | 0.67 | 0.06 |
| S3 | Predator:Nutrient | 1 | 0.0045 | 0.0045 | 2.5 | 184 | 0.11 | 160 | 0.11 | 0.79 |
| S4 | Predator | 1 | 0.6302 | 0.6302 | 154.3 | 185 | **0.00** | 161 | **0.00** | 27.01 |
| S4 | Nutrient | 1 | 0.1731 | 0.1731 | 42.4 | 185 | **0.00** | 161 | **0.00** | 7.42 |
| S4 | Predator:Nutrient | 1 | 0.4779 | 0.4779 | 117.0 | 185 | **0.00** | 161 | **0.00** | 20.49 |
| S5 | Predator | 1 | 0.0000 | 0.0000 | 0.8 | 176 | 0.37 | 152 | 0.37 | 0.25 |
| S5 | Nutrient | 1 | 0.0013 | 0.0013 | 36.7 | 176 | **0.00** | 152 | **0.00** | 11.28 |
| S5 | Predator:Nutrient | 1 | 0.0000 | 0.0000 | 0.1 | 176 | 0.80 | 152 | 0.80 | 0.02 |
| S6 | Predator | 1 | 0.0060 | 0.0060 | 5.6 | 185 | **0.02** | 161 | **0.02** | 1.44 |
| S6 | Nutrient | 1 | 0.0376 | 0.0376 | 35.0 | 185 | **0.00** | 161 | **0.00** | 9.02 |
| S6 | Predator:Nutrient | 1 | 0.0011 | 0.0011 | 1.0 | 185 | 0.32 | 161 | 0.32 | 0.26 |
| S7 | Predator | 1 | 0.0000 | 0.0000 | 0.0 | 100 | 1.00 | 80 | 1.00 | 0.00 |
| S7 | Nutrient | 1 | 0.0000 | 0.0000 | 2.2 | 100 | 0.14 | 80 | 0.14 | 0.22 |
| S7 | Predator:Nutrient | 1 | 0.0000 | 0.0000 | 0.4 | 100 | 0.55 | 80 | 0.55 | 0.03 |
| S8 | Predator | 1 | 0.0000 | 0.0000 | 2.7 | 101 | 0.10 | 81 | 0.11 | 1.71 |
| S8 | Nutrient | 1 | 0.0000 | 0.0000 | 25.4 | 101 | **0.00** | 81 | **0.00** | 16.25 |
| S8 | Predator:Nutrient | 1 | 0.0000 | 0.0000 | 1.1 | 101 | 0.29 | 81 | 0.29 | 0.73 |
| S9 | Predator | 1 | 0.0000 | 0.0000 | 0.3 | 100 | 0.57 | 79 | 0.57 | 0.27 |
| S9 | Nutrient | 1 | 0.0001 | 0.0001 | 18.4 | 100 | **0.00** | 79 | **0.00** | 15.45 |
| S9 | Predator:Nutrient | 1 | 0.0000 | 0.0000 | 0.4 | 100 | 0.52 | 79 | 0.52 | 0.36 |
| S10 | Predator | 1 | 0.0001 | 0.0001 | 16.1 | 161 | **0.00** | 137 | **0.00** | 7.99 |
| S10 | Nutrient | 1 | 0.0001 | 0.0001 | 13.1 | 161 | **0.00** | 137 | **0.00** | 6.50 |
| S10 | Predator:Nutrient | 1 | 0.0000 | 0.0000 | 5.9 | 161 | **0.02** | 137 | **0.02** | 2.95 |
| S11 | Predator | 1 | 0.0000 | 0.0000 | 0.8 | 141 | 0.37 | 117 | 0.37 | 0.48 |
| S11 | Nutrient | 1 | 0.0000 | 0.0000 | 8.4 | 141 | **0.00** | 117 | **0.00** | 5.03 |
| S11 | Predator:Nutrient | 1 | 0.0000 | 0.0000 | 1.8 | 141 | 0.19 | 117 | 0.19 | 1.05 |
| S12 | Predator | 1 | 0.0353 | 0.0353 | 12.2 | 185 | **0.00** | 161 | **0.00** | 2.19 |
| S12 | Nutrient | 1 | 0.2516 | 0.2516 | 87.0 | 185 | **0.00** | 161 | **0.00** | 15.62 |
| S12 | Predator:Nutrient | 1 | 0.0096 | 0.0096 | 3.3 | 185 | 0.07 | 161 | 0.07 | 0.60 |
| S13 | Predator | 1 | 0.0000 | 0.0000 | 0.0 | 184 | 0.89 | 160 | 0.89 | 0.01 |
| S13 | Nutrient | 1 | 0.0401 | 0.0401 | 16.4 | 184 | **0.00** | 160 | **0.00** | 6.30 |
| S13 | Predator:Nutrient | 1 | 0.0033 | 0.0033 | 1.3 | 184 | 0.25 | 160 | 0.25 | 0.51 |
| S14 | Predator | 1 | 0.0000 | 0.0000 | 0.0 | 117 | 0.88 | 95 | 0.89 | 0.02 |
| S14 | Nutrient | 1 | 0.0000 | 0.0000 | 18.6 | 117 | **0.00** | 95 | **0.00** | 13.65 |
| S14 | Predator:Nutrient | 1 | 0.0000 | 0.0000 | 0.1 | 117 | 0.81 | 95 | 0.81 | 0.04 |
| S15 | Predator | 1 | 0.0428 | 0.0428 | 24.0 | 185 | **0.00** | 161 | **0.00** | 4.94 |
| S15 | Nutrient | 1 | 0.0262 | 0.0262 | 14.7 | 185 | **0.00** | 161 | **0.00** | 3.03 |
| S15 | Predator:Nutrient | 1 | 0.0070 | 0.0070 | 3.9 | 185 | **0.05** | 161 | **0.05** | 0.81 |
| S16 | Predator | 1 | 0.0001 | 0.0001 | 0.0 | 185 | 0.91 | 161 | 0.91 | 0.00 |
| S16 | Nutrient | 1 | 2.0768 | 2.0768 | 360.0 | 185 | **0.00** | 161 | **0.00** | 44.80 |
| S16 | Predator:Nutrient | 1 | 0.0003 | 0.0003 | 0.1 | 185 | 0.81 | 161 | 0.81 | 0.01 |
| S18 | Predator | 1 | 0.3748 | 0.3748 | 40.2 | 185 | **0.00** | 161 | **0.00** | 4.11 |
| S18 | Nutrient | 1 | 0.0664 | 0.0664 | 7.1 | 185 | **0.01** | 161 | **0.01** | 0.73 |
| S18 | Predator:Nutrient | 1 | 0.1103 | 0.1103 | 11.8 | 185 | **0.00** | 161 | **0.00** | 1.21 |
| S19 | Predator | 1 | 0.0000 | 0.0000 | 3.0 | 144 | **0.09** | 120 | **0.09** | 1.40 |
| S19 | Nutrient | 1 | 0.0000 | 0.0000 | 22.2 | 144 | **0.00** | 120 | **0.00** | 10.31 |
| S19 | Predator:Nutrient | 1 | 0.0000 | 0.0000 | 1.6 | 144 | 0.21 | 120 | 0.21 | 0.73 |
| S20 | Predator | 1 | 0.0000 | 0.0000 | 0.8 | 178 | 0.36 | 154 | 0.36 | 0.20 |
| S20 | Nutrient | 1 | 0.0021 | 0.0021 | 56.9 | 178 | **0.00** | 154 | **0.00** | 13.52 |
| S20 | Predator:Nutrient | 1 | 0.0000 | 0.0000 | 0.2 | 178 | 0.66 | 154 | 0.66 | 0.05 |
| S21 | Predator | 1 | 0.0000 | 0.0000 | 2.4 | 112 | 0.13 | 93 | 0.13 | 2.06 |
| S21 | Nutrient | 1 | 0.0000 | 0.0000 | 0.6 | 112 | 0.43 | 93 | 0.43 | 0.55 |
| S21 | Predator:Nutrient | 1 | 0.0000 | 0.0000 | 0.4 | 112 | 0.55 | 93 | 0.55 | 0.32 |
| S22 | Predator | 1 | 0.0010 | 0.0010 | 0.3 | 182 | 0.57 | 158 | 0.57 | 0.09 |
| S22 | Nutrient | 1 | 0.1275 | 0.1275 | 41.5 | 182 | **0.00** | 158 | **0.00** | 11.37 |
| S22 | Predator:Nutrient | 1 | 0.0006 | 0.0006 | 0.2 | 182 | 0.65 | 158 | 0.65 | 0.06 |
| S23 | Predator | 1 | 0.0016 | 0.0016 | 0.4 | 185 | 0.53 | 161 | 0.53 | 0.10 |
| S23 | Nutrient | 1 | 0.0076 | 0.0076 | 1.9 | 185 | 0.17 | 161 | 0.17 | 0.49 |
| S23 | Predator:Nutrient | 1 | 0.0003 | 0.0003 | 0.1 | 185 | 0.79 | 161 | 0.79 | 0.02 |
| S24 | Predator | 1 | 0.0001 | 0.0001 | 0.2 | 183 | 0.64 | 159 | 0.64 | 0.09 |
| S24 | Nutrient | 1 | 0.0037 | 0.0037 | 9.0 | 183 | **0.00** | 159 | **0.00** | 3.95 |
| S24 | Predator:Nutrient | 1 | 0.0000 | 0.0000 | 0.1 | 183 | 0.82 | 159 | 0.82 | 0.02 |

Table S7: Statistical results of pairwise non-parametric linear mixed models testing differences in the relationship between relative abundance in the control and reduction in relative abundance by predation for different nutrient concentrations between the categories with 0.0001 as the reference nutrient concentration.

|  | 0.0001-0.001 | | 0.0001-0.01 | | 0.0001-0.1 | |
| --- | --- | --- | --- | --- | --- | --- |
| Categories | **t** | **p** | **t** | **p** | **t** | **p** |
| Common/Fast:Common/Slow | 1.6 | 0.11 | -4.2 | <0.01* | -2.8 | <0.01* |
| Common/Fast:Rare/Fast | -1,0 | 0.32 | -1.4 | 0.16 | -0.2 | 0.86 |
| Common/Fast:Rare/Slow | 0.4 | 0.57 | -3.6 | <0.01* | 0.4 | 0.67 |
| Common/Slow: Rare/Slow | -1.4 | 0.16 | 2.1 | 0.04* | 4.6 | <0.01* |
| Common/Slow: Rare/Fast | -1.9 | 0.06 | 1.5 | 0.13 | 1.7 | 0.08 |
| Rare/Fast: Rare/Slow | 1.6 | 0.11 | -1.1 | 0.29 | 1.0 | 0.33 |

Table S8: statistical results of pairwise linear models testing differences in the relationship between relative abundance in the control and reduction in relative abundance by predation between categories over all nutrient levels; data analysis after exclusion of taxon S4, therefore only group Rare/Slow is shown; for all results see Table S9.

| Categories | t | p |
| --- | --- | --- |
| Common/Fast:Common/Slow | -5.9 | <0.01* |
| Common/Fast:Rare/Fast | -12.8 | <0.01* |
| Common/Fast:Rare/Slow | 0.9 | 0.35 |
| Common/Slow: Rare/Slow | -15.7 | <0.01* |

Table S9: Statistical results of pairwise linear models resting differences in the relationship between relative abundance in the control and reduction in relative abundance by predation for different nutrient concentrations between the groups with 0.0001 as the reference nutrient concentration; data analysis after exclusion of taxon S4, therefore only category Rare/Slow is shown; for all results see Table S10.

|  | 0.0001-0.001 | | 0.0001-0.01 | | 0.0001-0.01 | |
| --- | --- | --- | --- | --- | --- | --- |
| Categories | **t** | **p** | **t** | **p** | **t** | **p** |
| Rare/Slow:Common/Fast | 1.5 | 0.13 | 4.0 | <0.01* | 0.7 | 0.49 |
| Rare/Slow:Common/Slow | 3.8 | <0.01* | -1.6 | 0.11 | -3.5 | <0.01* |
| Rare/Slow:Rare/Fast | 0.1 | 0.95 | 1.2 | 0.24 | 0.4 | 0.72 |
|  |  |  |  |  |  |  |


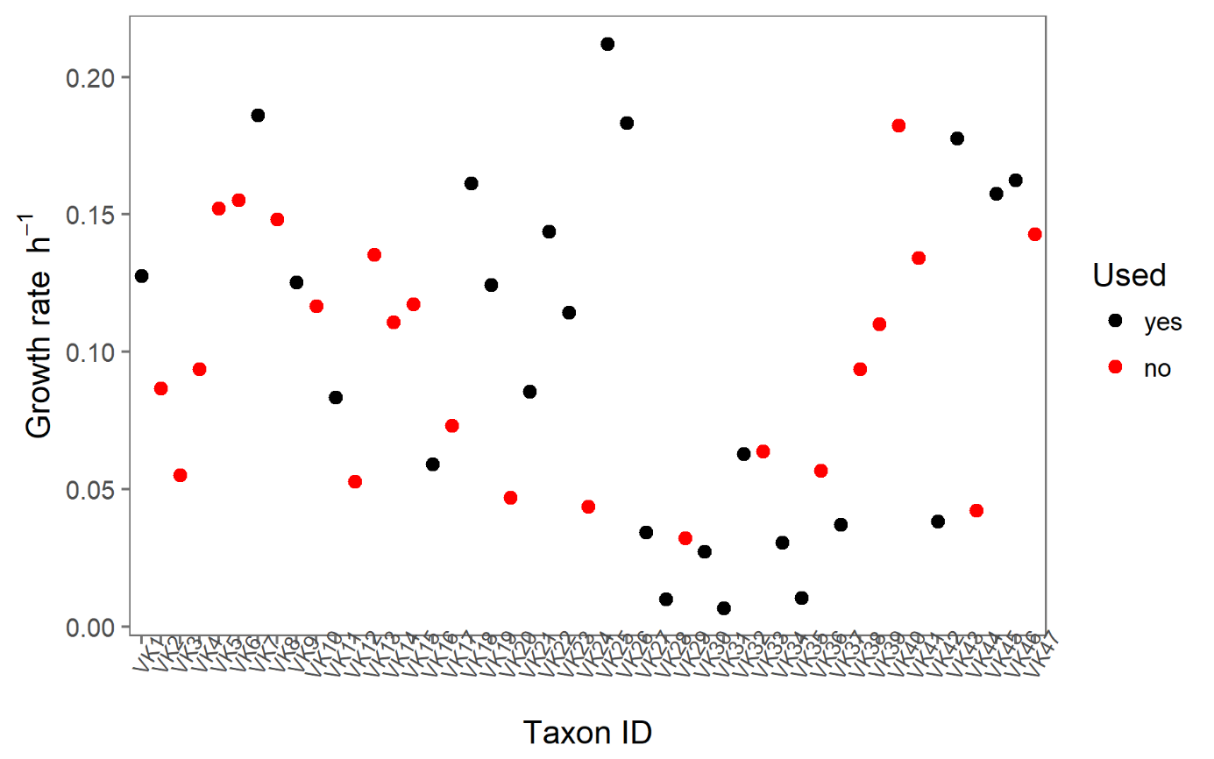


Fig. S1: Distribution of growth rates (h^-1^) of all bacterial isolates in the isolate collection; isolates used in this study are marked in red.


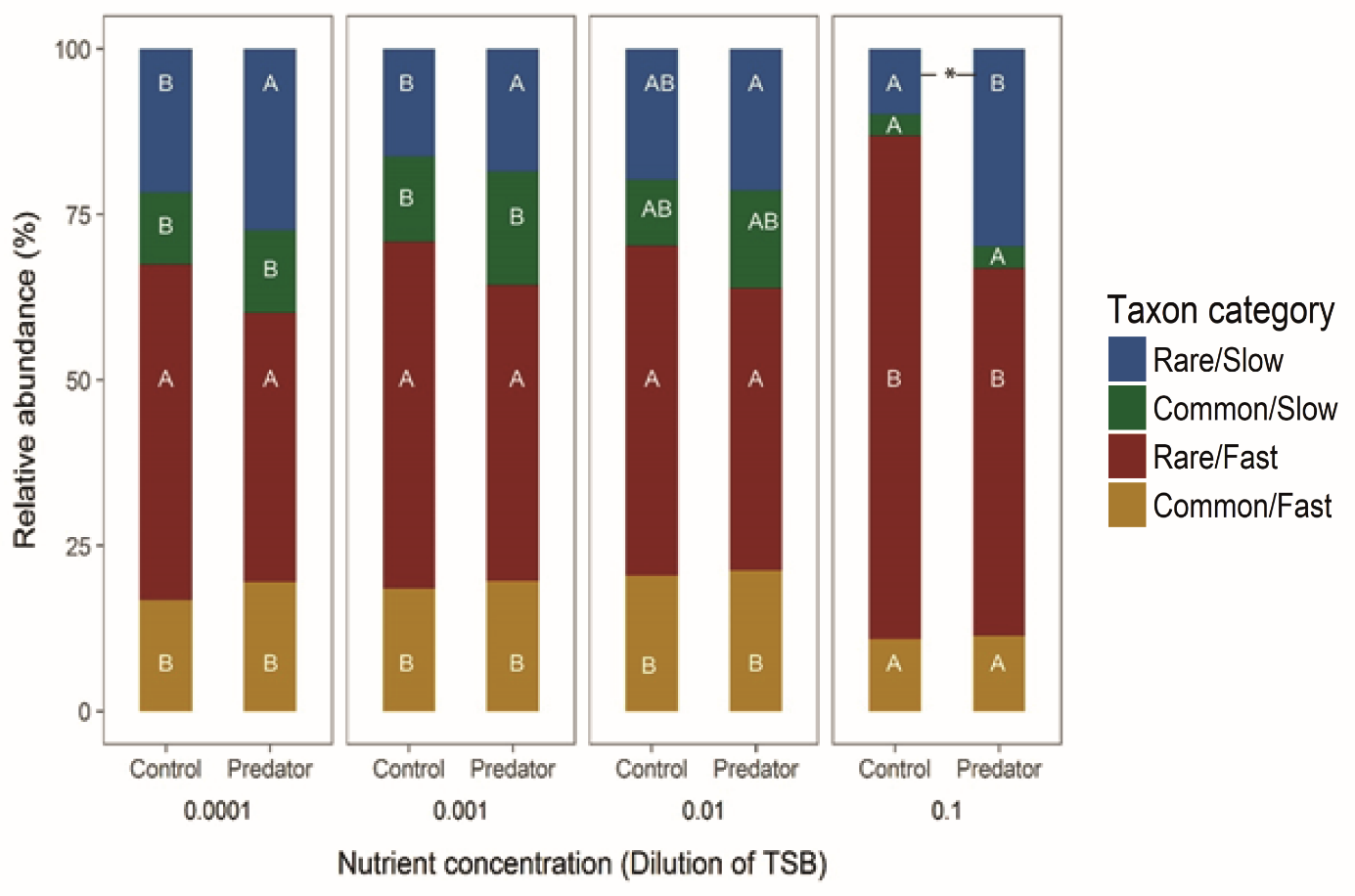


Fig. S2: Average relative abundance of the four bacterial taxon categories at the different nutrient concentrations in the predator free control and the predation treatment; different letters indicate significant differences between nutrient concentrations within one trait group; significant differences are marked with *.


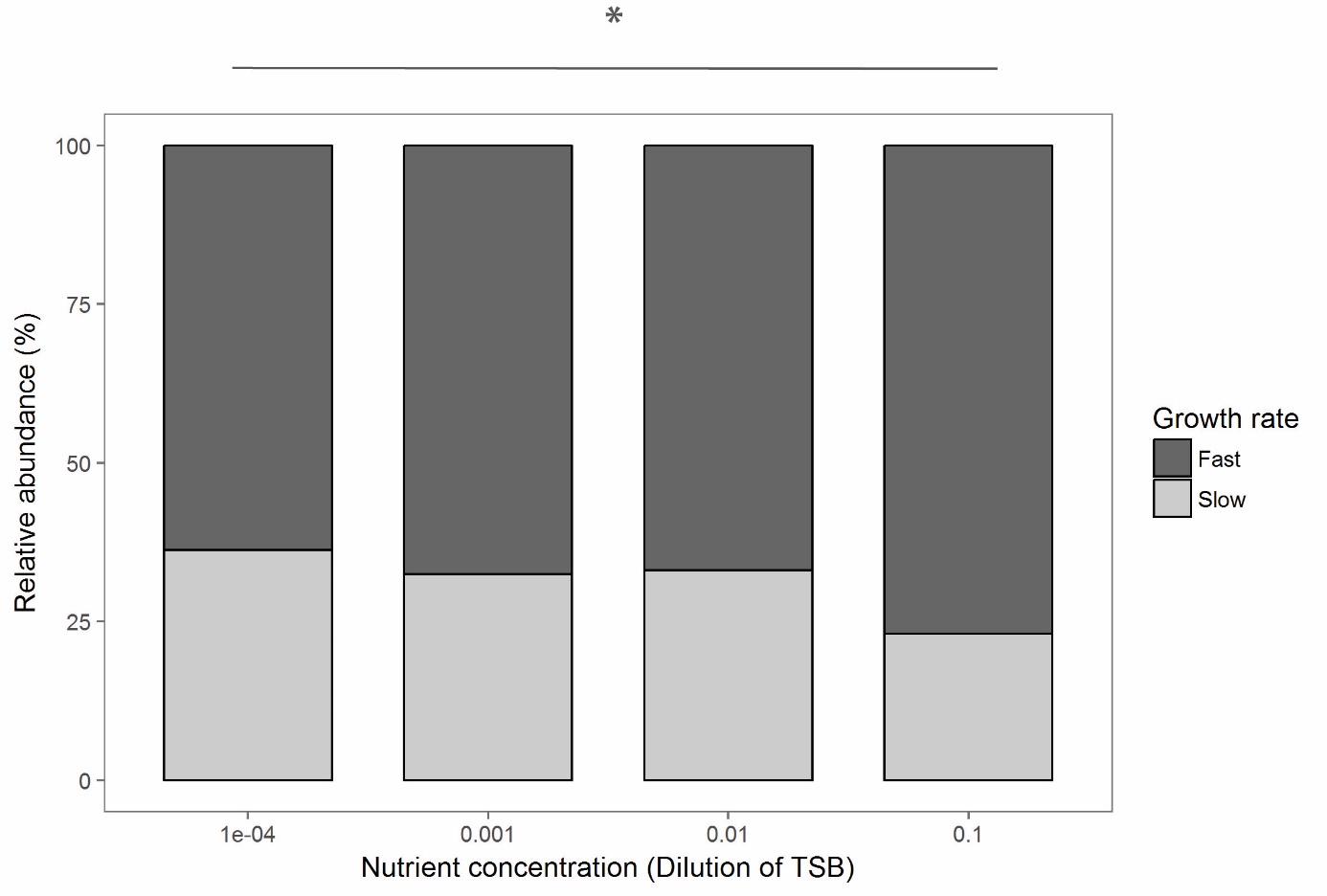


Figure S3: Average relative abundance of bacterial taxa with potential fast and slow growth rates at the four different nutrient concentrations; relative abundances are averaged over predation treatment, community and abundance categories; significant linear relationships of abundance with nutrient concentration are indicated with *.


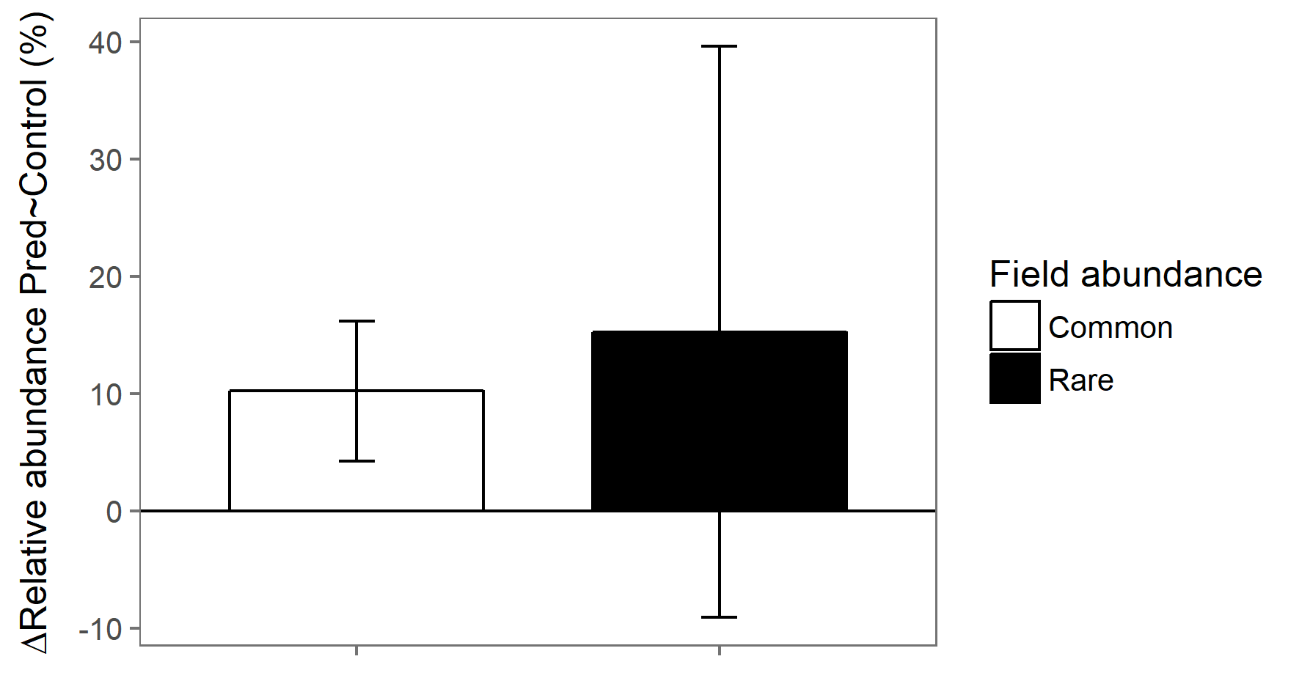


Fig. S4: Average percentage change in bacterial relative abundance between the control and the predation treatment concentrations for species that were characterized as common or rare in the field; results are averaged over nutrient concentration, community and potential growth rate; error bars represent the standard error.


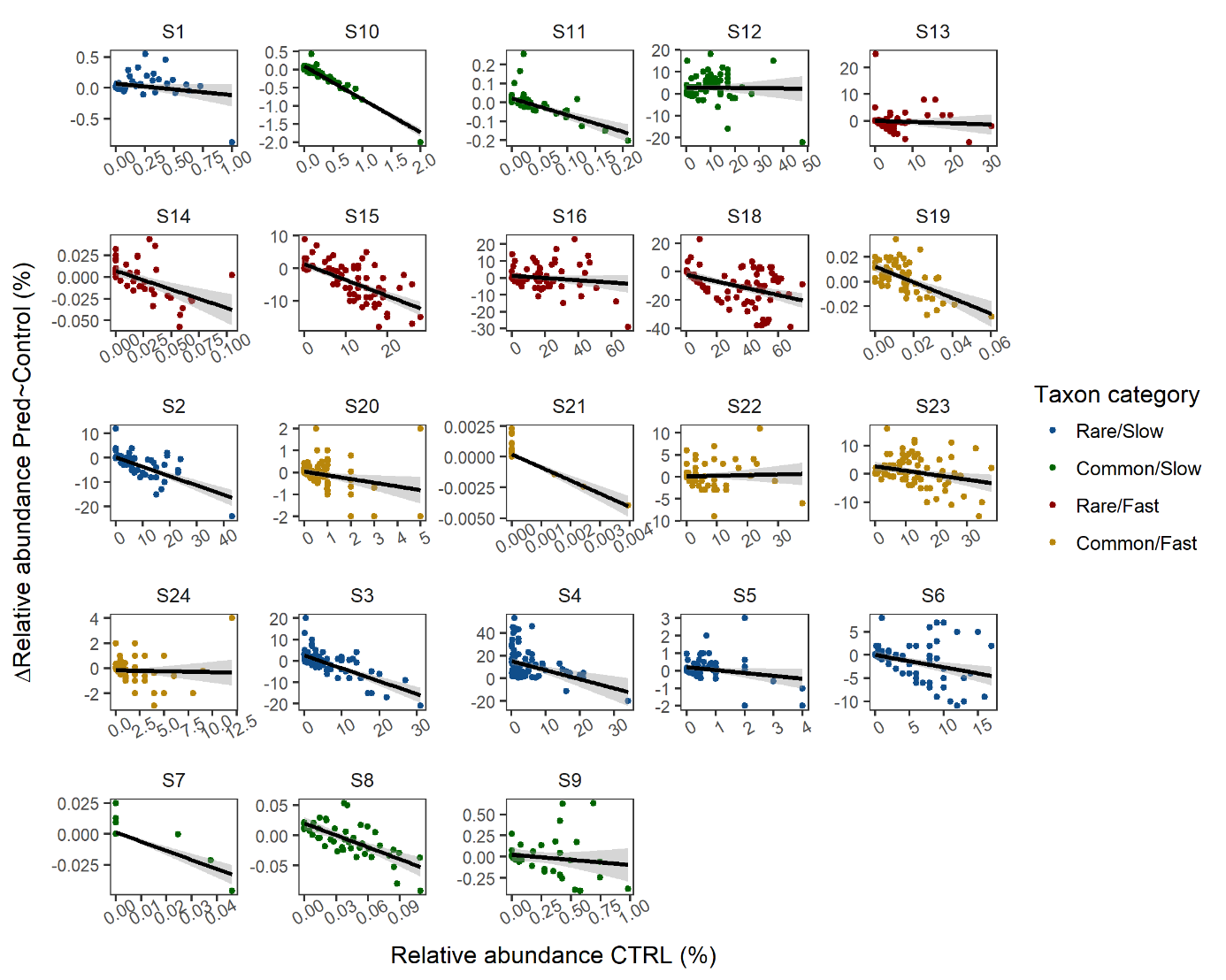


Figure S5: Abundance loss in the predation treatment plotted against relative abundance in the control for each taxon; each dot represents one community at the 4 nutrient levels; different colours represent the four taxon categories; lines represent a linear model fit for each taxon.


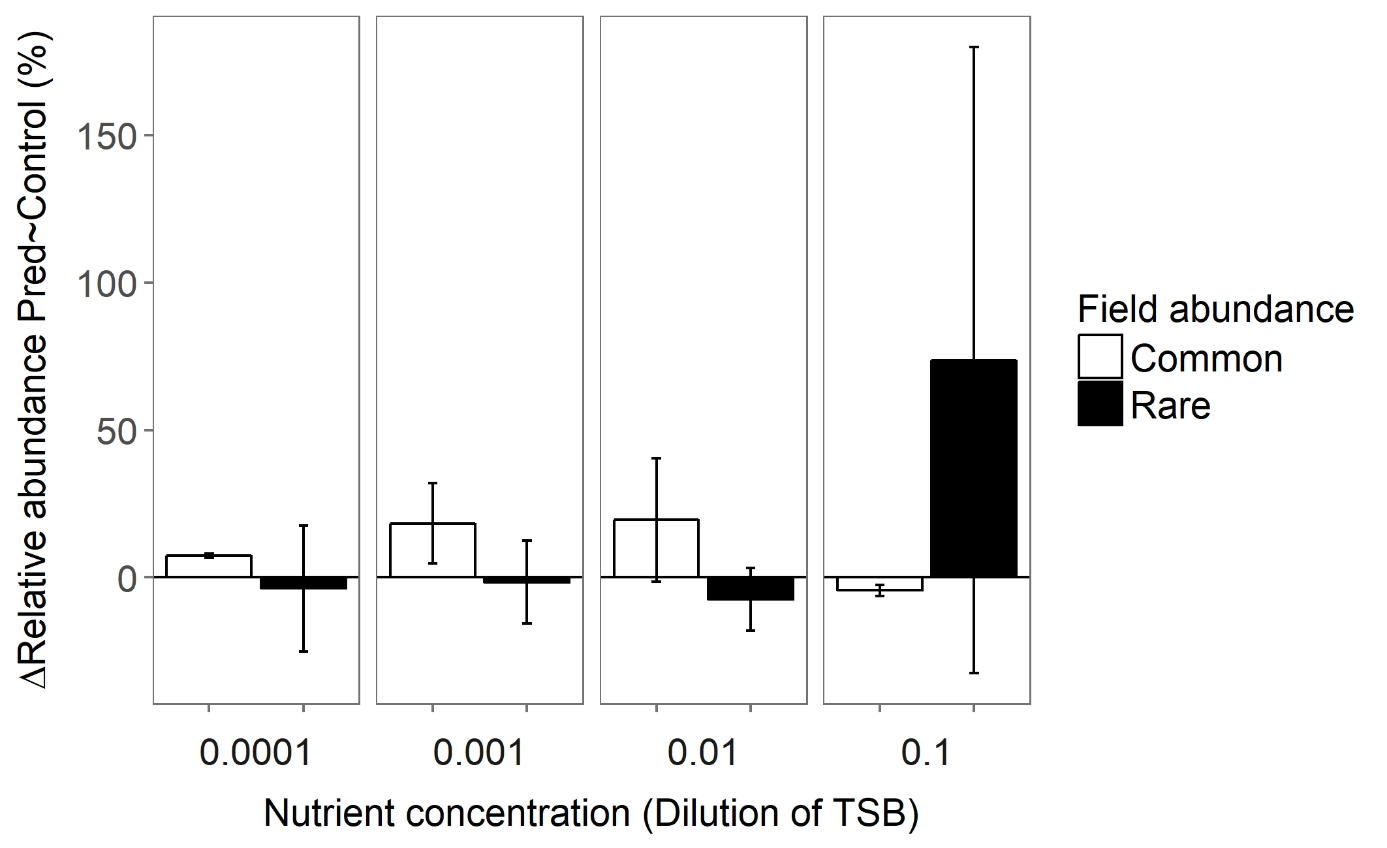


Figure S6: Average percentage change in relative abundance between the control and the predation treatment at the four different nutrient concentrations for taxa that were characterized as common or rare in the field; results are averaged over community and potential growth rate; error bars represent the standard error.


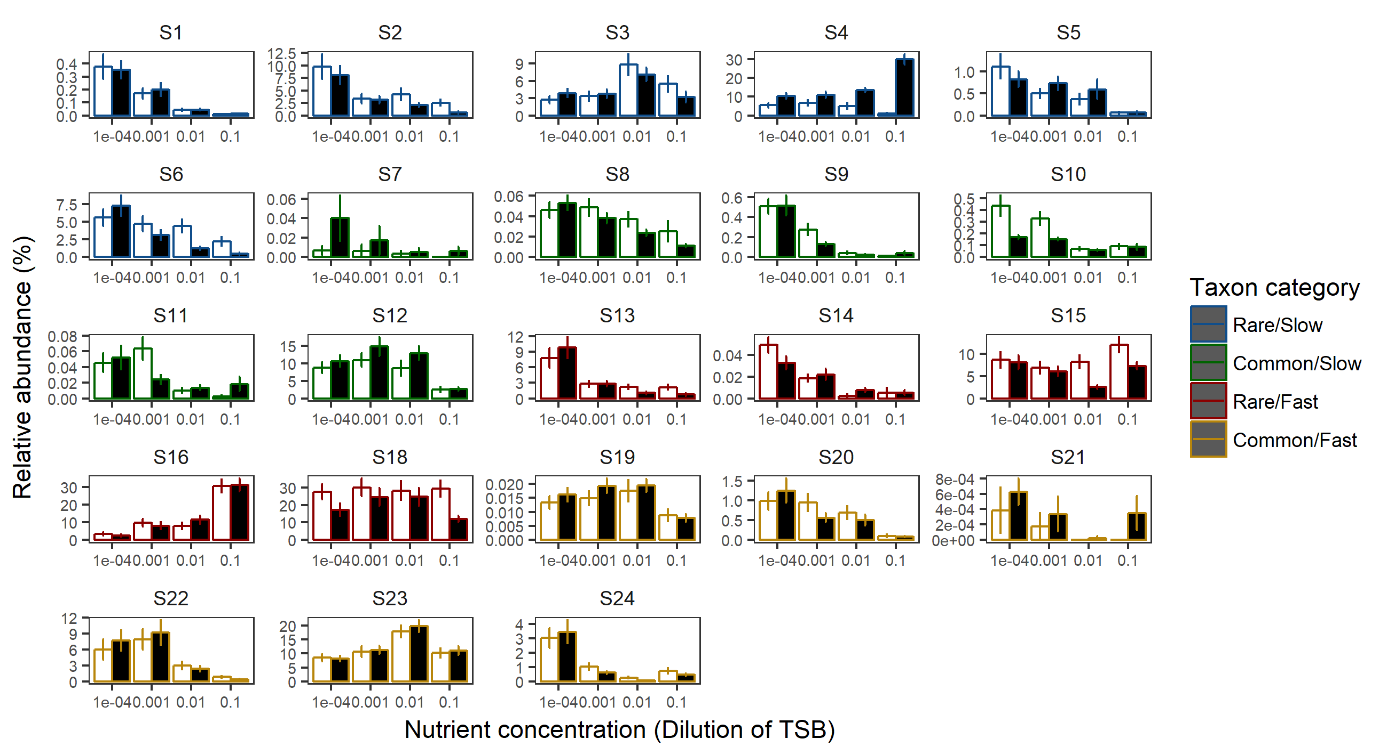


Figure S7: Average relative abundance of each taxon at the different nutrient concentrations and without (white bars) and with (black bars) predation; relative abundances were averaged over communities; errorbars indicate the standard error; different colours indicate the four taxon categories.


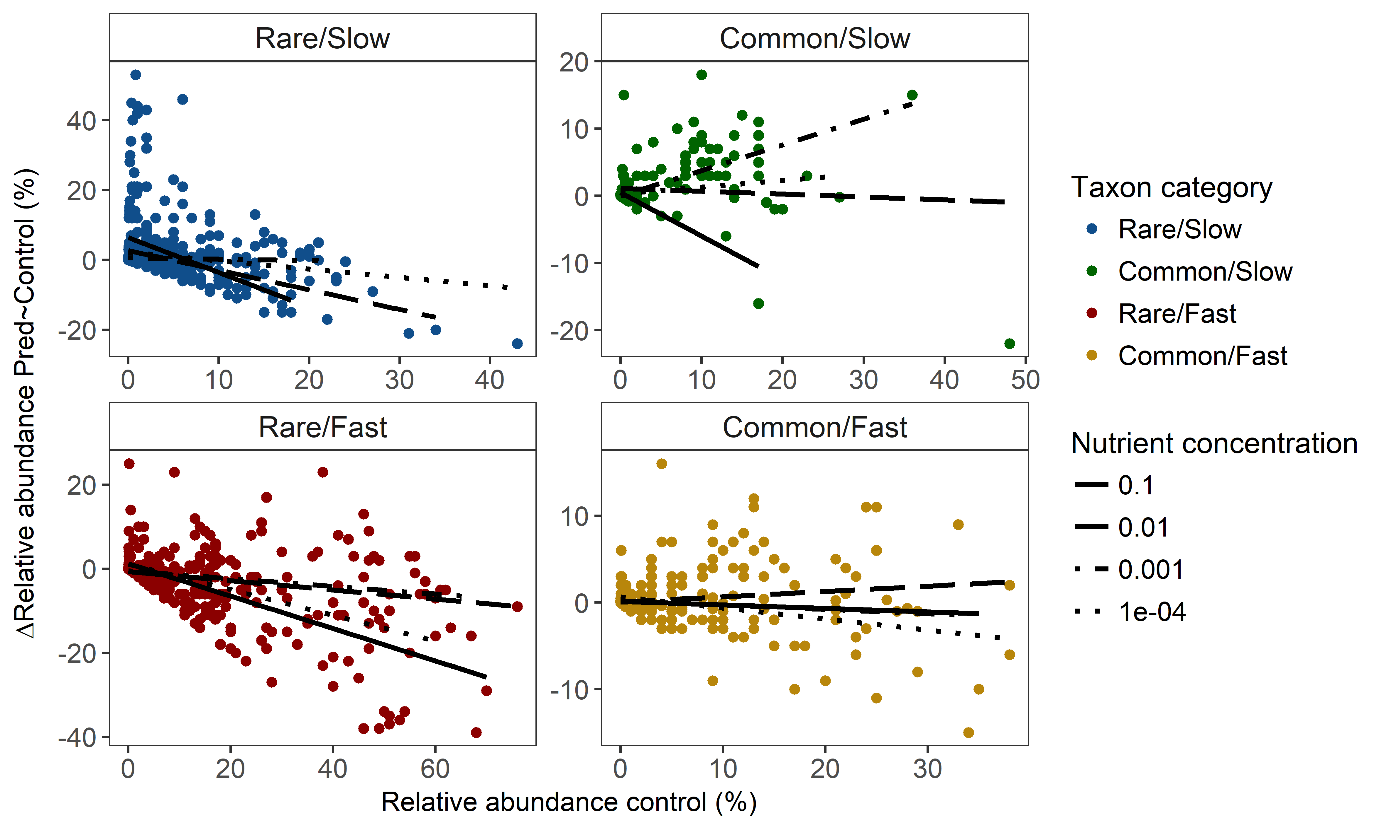


Figure S8: Linear model fit of the relationship between relative abundance in the control and relative abundance reduction in the predation treatment; dots represent the individual taxa in each community; different colours represent the four taxon categories.


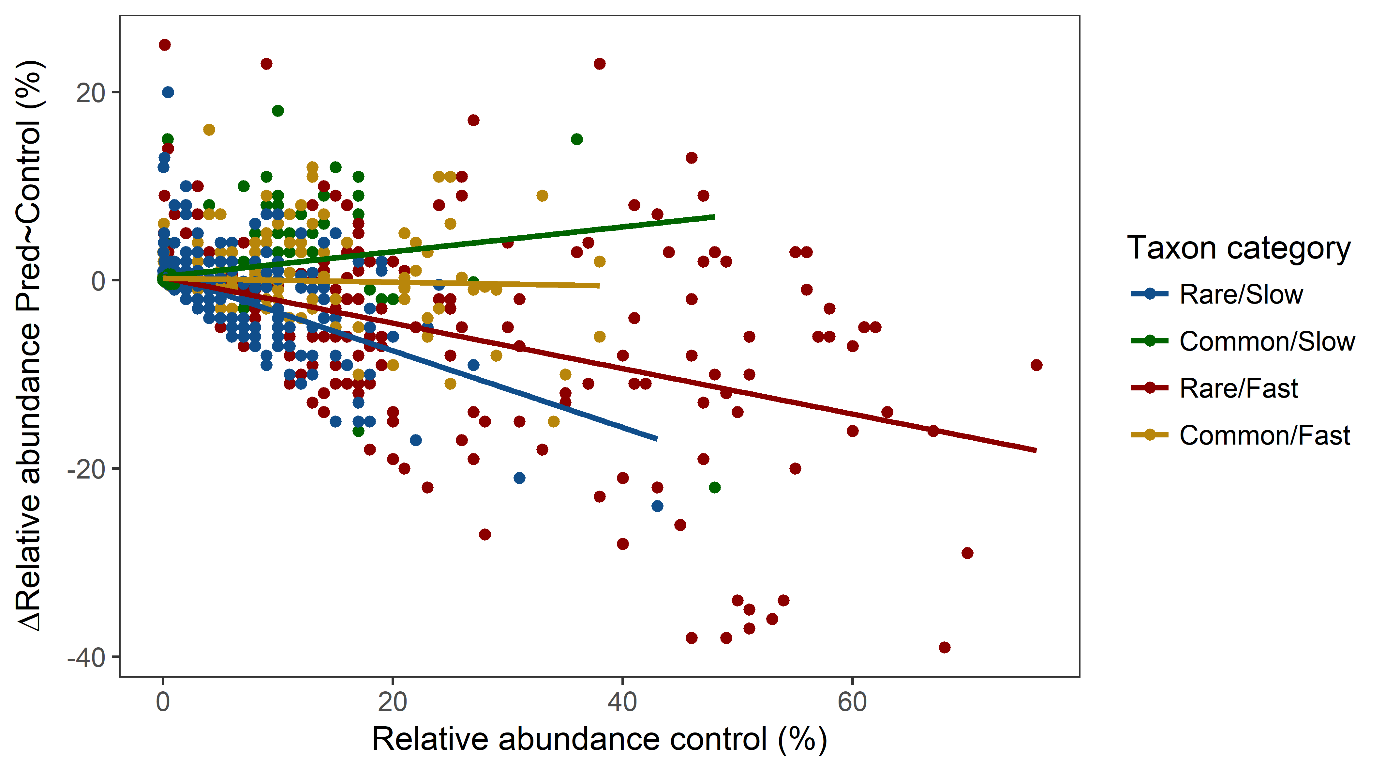


Figure S9: Linear model fit with standard error of the relationship between relative abundance in the control and relative abundance reduction in the predation treatment after omitting taxon S4; dots represent the individual taxa in each community; different colours represent the four taxon categories.


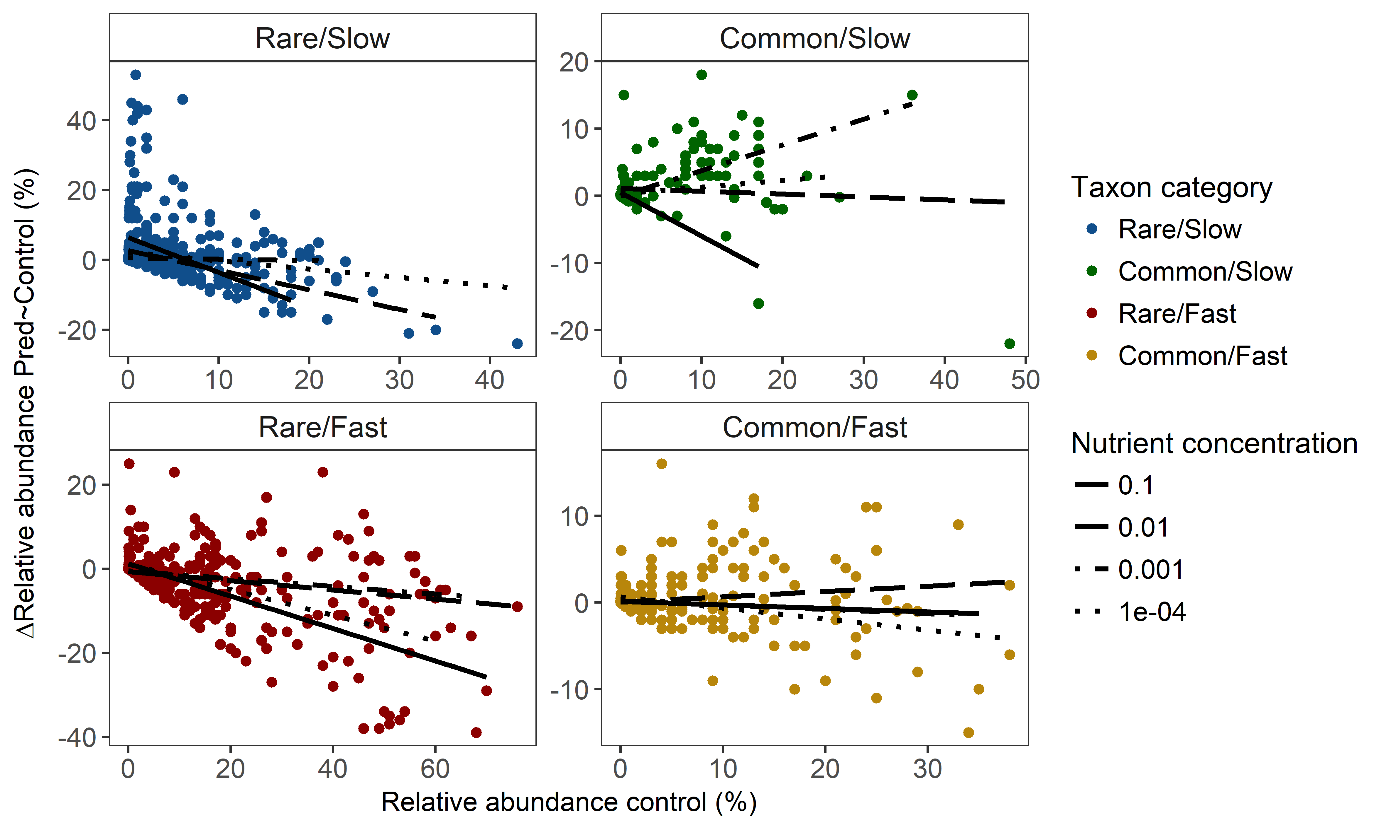


Figure S10: Linear model fit of the relationship between relative abundance in the control and relative abundance reduction in the predation treatment at the four different nutrient concentrations; dots represent the individual taxa in each community; different colours represent the four taxon categories and different linetypes represent the different nutrient concentrations.


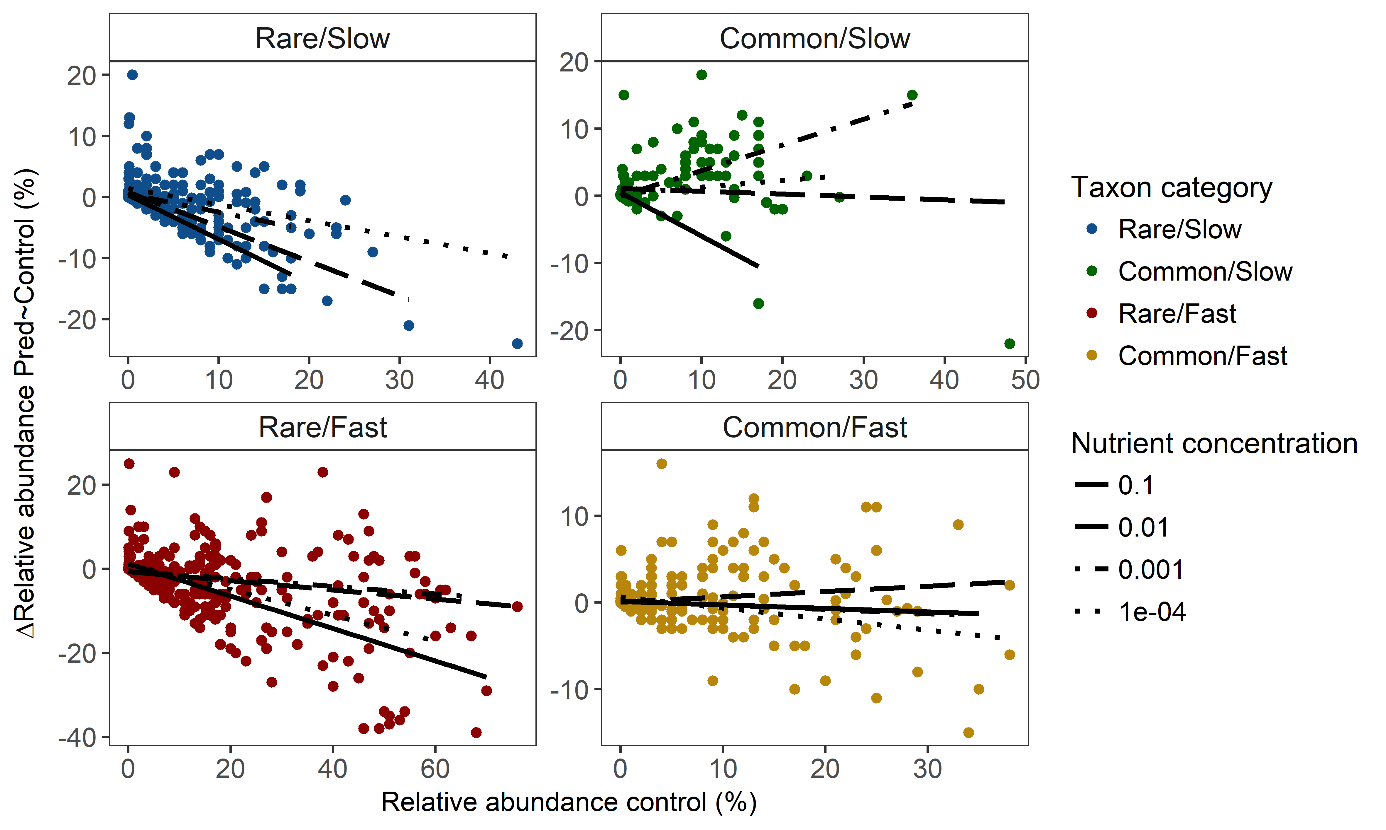


Figure S11: Linear model fit with standard error of the relationship between relative abundance in the control and relative abundance reduction in the predation treatment at the four different nutrient concentrations after omitting taxon S4; dots represent the individual taxa in each community; different colours represent the four taxon categories and different linetypes represent the different nutrient concentrations.


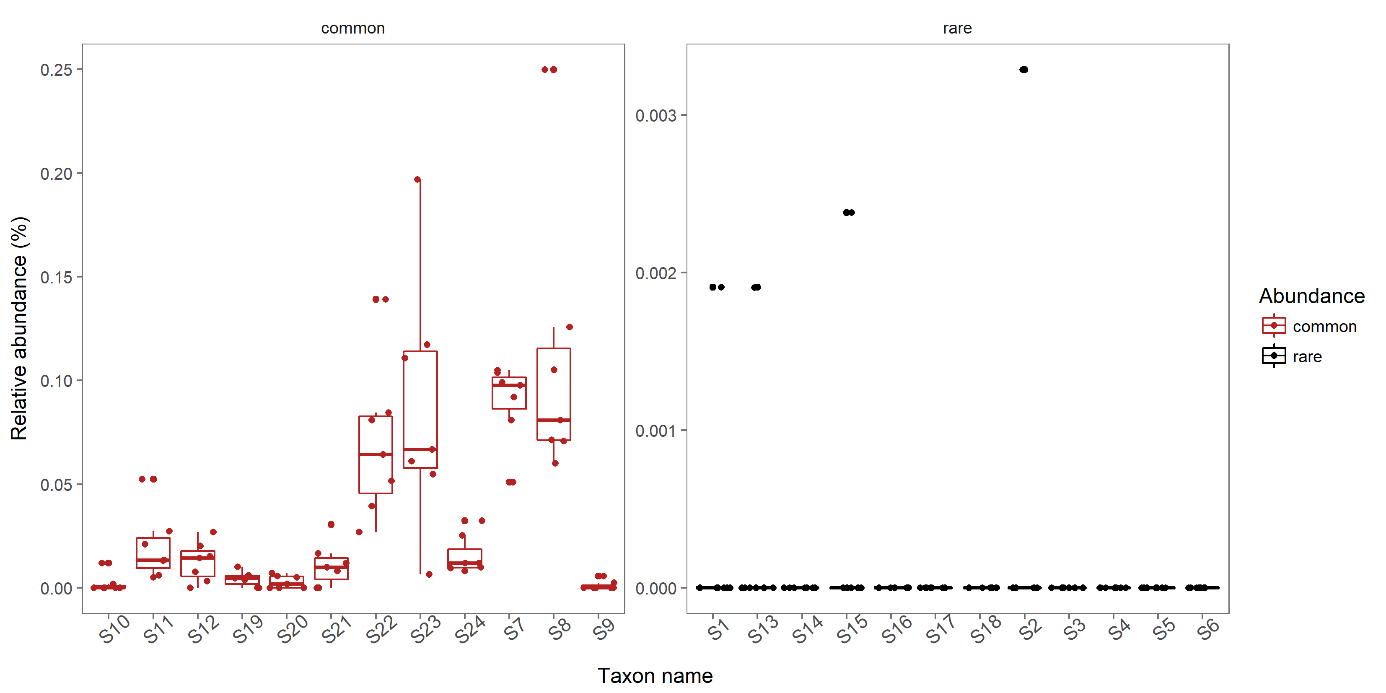


Figure S12: Boxplot of relative abundances of taxa in the field in the seven soil samples used for construction of the sequencing database. Each dot represents the abundance of the respective taxon in one soil sample.

Geisen, S., et al. (2014). "Two new species of the genus Stenamoeba (Discosea, Longamoebia): Cytoplasmic MTOC is present in one more amoebae lineage." European Journal of Protistology **50**(2): 153-165.

Kurm, V., et al. (2017). "Low abundant soil bacteria can be metabolically versatile and fast growing." Ecology **98**(2): 555-564.
